# Spatial and spectral trajectories in typical neurodevelopment from childhood to middle age

**DOI:** 10.1162/netn_a_00077

**Published:** 2019-03-01

**Authors:** Benjamin A. E. Hunt, Simeon M. Wong, Marlee M. Vandewouw, Matthew J. Brookes, Benjamin T. Dunkley, Margot J. Taylor

**Affiliations:** Department of Diagnostic Imaging, The Hospital for Sick Children, Toronto, Canada; Neurosciences and Mental Health Program, The Hospital for Sick Children Research Institute, Toronto, Canada; Department of Diagnostic Imaging, The Hospital for Sick Children, Toronto, Canada; Neurosciences and Mental Health Program, The Hospital for Sick Children Research Institute, Toronto, Canada; Department of Diagnostic Imaging, The Hospital for Sick Children, Toronto, Canada; Neurosciences and Mental Health Program, The Hospital for Sick Children Research Institute, Toronto, Canada; The Sir Peter Mansfield Imaging Centre, School of Physics and Astronomy, University of Nottingham, Nottingham, United Kingdom; Department of Diagnostic Imaging, The Hospital for Sick Children, Toronto, Canada; Neurosciences and Mental Health Program, The Hospital for Sick Children Research Institute, Toronto, Canada; Department of Medical Imaging, University of Toronto, Toronto, Canada; Department of Diagnostic Imaging, The Hospital for Sick Children, Toronto, Canada; Neurosciences and Mental Health Program, The Hospital for Sick Children Research Institute, Toronto, Canada; Department of Psychology, University of Toronto, Toronto, Canada; Department of Medical Imaging, University of Toronto, Toronto, Canada

**Keywords:** Neurodevelopment, MEG, Functional connectivity, Phase synchronisation, Power spectral density, Maturational trajectories, Resting state

## Abstract

Detailed characterization of typical human neurodevelopment is key if we are to understand the nature of mental and neurological pathology. While research on the cellular processes of neurodevelopment has made great advances, in vivo human imaging is crucial to understand our uniquely human capabilities, as well as the pathologies that affect them. Using magnetoencephalography data in the largest normative sample currently available (324 participants aged 6–45 years), we assess the developmental trajectory of resting-state oscillatory power and functional connectivity from childhood to middle age. The maturational course of power, indicative of local processing, was found to both increase and decrease in a spectrally dependent fashion. Using the strength of phase-synchrony between parcellated regions, we found significant linear and nonlinear (quadratic and logarithmic) trajectories to be characterized in a spatially heterogeneous frequency-specific manner, such as a superior frontal region with linear and nonlinear trajectories in theta and gamma band respectively. Assessment of global efficiency revealed similar significant nonlinear trajectories across all frequency bands. Our results link with the development of human cognitive abilities; they also highlight the complexity of neurodevelopment and provide quantitative parameters for replication and a robust footing from which clinical research may map pathological deviations from these typical trajectories.

## Author Summary

Imagine that we could predict which children will go on to develop depression or anxiety disorders using functional brain imaging? This would allow early intervention and, in some, negation of the illness. The first step toward this goal is understanding the neurodevelopmental trajectory of healthy individuals. We can then recognize the developmental deviations with specific illnesses.

We used the largest developmental MEG dataset available (*n* = 324) to characterize the developmental trajectories of functional connectivity in a cohort aged 6–45 years. We find evidence for nonlinear trajectories in two graph theory metrics, heterogeneously defined in brain space with opposing trajectories in different oscillatory frequencies. Our findings highlight the intricacy of typical neurodevelopment and provide foundational quantifications for replication in different modalities and application to clinical cohorts.

## INTRODUCTION

Compared with other mammalian species, human neonates are relatively disadvantaged at birth. While giraffe calves are able to walk within an hour of birth and run within a day (Kaleta & Marczewska, 2007), it takes human neonates more than 1 year to walk unaided, and many months thereafter to run (Størvold, Aarethun, & Bratberg, 2013). Nonetheless, as children, humans develop uniquely complex cognitive abilities and motor control. Protracted ex utero neurodevelopmental processes continue well into adulthood (Tottenham, 2014), and they are characterized in early life by simultaneous apoptosis, synaptogenesis, and myelination of axons (Giedd, 1999; Tau & Peterson, 2010). Positron emission tomography (PET) studies indicate that overall brain metabolism rises to twice that of adults in 4- to 5-year-olds, and remains constant at that level until almost 10 years of age (Chugani, Phelps, & Mazziotta, 1987), which is thought to indicate the energy demands of these processes. Oligodendrocyte-based development continues into adulthood, with cortical myelination continuing to increase until the third decade of life (Shafee, Buckner, & Fischl, 2015) and white matter volumes peaking in the fifth decade of life (Paus et al., 2001). Healthy neurodevelopment ex utero is highly complex, with different processes following regionally specific, often nonlinear, trajectories ranging in time span from years to decades. Characterizing typical developmental trajectories is key if we are to understand why some children develop pathology, such as mental disorders or neurological conditions, while others remain free from illness. While we have a rapidly increasing comprehension of neurodevelopment at a cellular level (e.g., Tau & Peterson, 2010), a more modest literature offers descriptions of human neurodevelopment derived from in vivo measurements. Understanding at this level is crucial, given that the symptomology of many disorders is reflected in the impairment of complex and uniquely human cognition and behavior. Here we characterize typical neurodevelopment using in vivo neurophysiology in a large normative cohort.

Functional magnetic resonance imaging (fMRI) and electroencephalography (EEG) have made advances in our conception of human neurodevelopment. However, fMRI depends on the blood oxygenation level dependent signal, which has a variable lag between neural recruitment and signal change in the order of seconds (Yeşilyurt, Whittingstall, Uǧurbil, Logothetis, & Uludaǧ, 2010). Further, recent improvements in fMRI (pre)processing have called many “classic” developmental fMRI findings into question, particularly the many studies that failed to adequately account for head motion–induced artefacts (Grayson & Fair, 2017). EEG offers a direct measurement of electrophysiology but because of difficulties in modeling the inhomogeneous and geometrically complex conductivity profile in the head, the spatial resolution is limited. Therefore, a more promising technique is magnetoencephalography (MEG), which offers a noninvasive and direct measurement of in vivo brain function. Measuring the minute band-limited magnetic fields generated by synchronous postsynaptic potentials in populations of pyramidal neurons (Hämäläinen, Hari, Ilmoniemi, Knuutila, & Lounasmaa, 1993), MEG measurements are on the order of milliseconds and, when combined with appropriately implemented spatial filtering informed by anatomical MRI, offer a millimeter spatial resolution (Troebinger, López, Lutti, Bestmann, & Barnes, 2014). In combination, these features make MEG ideally suited to the characterization of neurodevelopment (Lopes da Silva, 2013), both in terms of fundamental electrophysiological features and in terms of functional connectivity between disparate regions.

Neural oscillations, as measured by (M)EEG, are integral to healthy brain function (Fries, 2005). Oscillations are characterized by their oscillatory power and phase, the latter thought to enable interregional communication (Fries, 2005). Despite being fundamental to the oscillation, the two measurements are minimally related and provide complementary insights into underlying neurophysiology. Here we investigated how both features change during typical neurodevelopment. Despite many decades of research with both EEG and MEG, a limited literature describes typical neurodevelopment from an electrophysiological perspective. The majority of these investigations used EEG with a small number of electrodes, allowing limited comparisons to MEG. Nonetheless, a consistent finding is that power spectral density (PSD), linked to local processing, decreases with age in the slower frequency bands (delta, ∼1–4 Hz, and theta, ∼4–7 Hz; Brookes et al., 2018; Gómez et al., 2017; Miskovic et al., 2015; Perone, Palanisamy, & Carlson, 2018; Vlahou, Thurm, Kolassa, & Schlee, 2014). There are also some reports of higher frequency PSD decreasing with age, such as alpha (∼8–13 Hz; Dias et al., 2015; Gómez et al., 2017) and beta (∼13–30 Hz; Rodriguez-Martinez, Barriga-Paulino, Rojas-Benjumea, & Gómez, 2015) frequencies. Whitford and colleagues (Whitford et al., 2007) collected EEG and structural MRI data in 10- to 30-year-old subjects. They found similar curvilinear relations between age and PSD and between age and cortical volume in frontal and parietal lobes. The authors suggested that PSD is related to synaptic pruning, with decreasing PSD in these lobes indicating decreasing synapse numbers. However, this relationship may not hold for all frequency bands, with other reports demonstrating an increase in PSD with age in alpha, beta, and gamma (∼30–70 Hz) bands (Benninger, Matthis, & Scheffner, 1984; Clarke, Barry, McCarthy, & Selikowitz, 2001; Perone et al., 2018).

While PSD is reported to be intimately linked to neurodevelopment, a more intuitive empirical focus is developmental changes in how brain regions communicate. Since the first observation of statistical dependency between isolated regions during rest (Biswal, Yetkin, Haughton, & Hyde, 1995), the field of functional connectivity (FC) has made great progress. Assessment of brain function in the absence of a task, often termed the resting state (Fox & Raichle, 2007), has proven to be a powerful technique for the study of neurodevelopment. Where a task may be more or less difficult for participants of different ages, the external stimuli during a resting-state acquisition is identical across the life span. Abnormal MEG-derived resting-state FC has been found in psychiatric conditions such as schizophrenia (e.g., Brookes et al., 2016), major depressive disorder (MDD; e.g., Nugent, Robinson, Coppola, Furey, & Zarate, 2015), and post-traumatic stress disorder (PTSD; e.g., Dunkley et al., 2015). In pediatric populations, reduced functional connectivity has been found in children diagnosed with autism spectrum disorder during a working memory task (Urbain et al., 2016) and in children born very preterm (Ye, AuCoin-Power, Taylor, & Doesburg, 2016). Given this growing evidence base, characterization of FC development across the life span in healthy subjects is a highly pertinent research area.

A limited literature has assessed the electrophysiological basis of neurodevelopment in terms of whole-brain functional connectivity changes across the life span. Schäfer and colleagues (Schäfer, Morgan, Ye, Taylor, & Doesburg, 2014) assessed whole-brain functional connectivity using MEG-derived amplitude envelope correlations in 59 participants aged 6 to 34 years. Using a priori defined canonical resting-state networks, they found global within- and between-network connectivity to linearly increase with age, in the theta, and most strikingly in the alpha and beta bands. However, a significant amount of evidence indicates cortical developmental trajectories to take a nonlinear form.

Studies assessing cortical thickness find development to take an inverted U, quadratic trajectory on a near global basis between the ages of 3 and 33 years (Giedd, 1999; Giedd & Rapoport, 2010; Gogtay et al., 2004; Shaw et al., 2008; Vandekar et al., 2015), although some also find evidence for logarithmic trajectories in a younger sample (1–6 years; Remer et al., 2017). Studies using fMRI to investigate neurotypical trajectories have also found this to be the case, such as Dosenbach et al. (2010) who found asymptotic development curves to be most successful at predicting brain age via machine learning in a sample aged between 7 and 30 years. Given the intimate relation between hemodynamic and electrophysiological techniques (see Hall, Robson, Morris, & Brookes, 2014, for review) and between electrophysiology and cortical structure (Hunt et al., 2016; Tewarie et al., 2016; Tewarie et al., 2014), nonlinear models are likely to be most successful in capturing electrophysiological maturation.

Whole-brain measures of FC offer great utility (Brookes et al., 2016) but can generate an unmanageable mass of data for each individual, leading many groups to adopt graph theory metrics that collapse across connectivity matrices. Here we used two such measures termed node strength and global efficiency (GE; Rubinov & Sporns, 2010; Sporns, 2011). Node strength is derived as the sum of all connections (edges) to a region (node), which measures how connected each region is within the greater network, whereas GE is a global measure assessing the networks’ ability to transmit information on a global scale and is influenced most by shorter paths (Rubinov & Sporns, 2010). Previously, GE was measured from resting MEG data, and was found to relate to functional coupling within networks (de Pasquale, Della Penna, Sporns, Romani, & Corbetta, 2016). Node strength has been derived consistently and used more widely than GE across numerous imaging modalities. In MEG, node strength measures are increased in PTSD (Dunkley et al., 2016) and reduced in mild traumatic brain injury (Pang, Dunkley, Doesburg, da Costa, & Taylor, 2016), indicating that this metric has sufficient sensitivity to detect differential functional perturbations in these behaviorally overlapping conditions. The developmental trajectory of node strength is somewhat contested within the MRI literature, with some studies reporting strength decreases between 7 and 22 years (Supekar, Musen, & Menon, 2009), some reporting age-related increases between 8 and 16 (J. R. Sato et al., 2014), and others characterizing strength using nonlinear analyses, reporting strength to take a negative quadratic form across a much larger age range (7 to 85 years; Cao et al., 2014). A recent MEG study found strength to develop nonlinearly between 9 and 25 years, with theta band strength following a quadratic developmental curve and alpha, beta, and gamma to take a monotonically increasing or decreasing form (Brookes et al., 2018). However, strength was averaged across all cortical parcels, precluding regional results and conclusions about topographic changes in strength.

Although GE has been used less frequently than node strength, a recent study by Kahn and colleagues (Khan et al., 2018) adopted nonlinear graph theory analyses. Using MEG-derived amplitude envelope connectivity (AEC) from a large sample (*n* = 162 participants) aged 7 to 29 years, the authors found significant nonlinear increases in GE in the gamma band only. Using machine learning based on a composite of graph metrics, the authors found linear increases in the beta band to best predict subject age, and, using a different graph metric composite, found the most successful predictors of age in the gamma band to take a quadratic (inverted U) form. In contrast to the study by Schäfer et al. (2014), the group did not find any significant effects in the alpha band.

Here we characterized neurodevelopmental connectivity, measured by MEG, in the largest developmental cohort that currently exists (*N* = 324, from a sample of 425). Because of the mixed MRI and MEG literature and our uniquely large sample size, we conducted an initial analysis of node strength, derived from measurements of phase synchrony between regions. Given the recent findings of Brookes and colleagues, we hypothesized that strength would be characterized predominantly by negative quadratic curves in theta, nonlinearly increasing curves in alpha and beta, and nonlinearly decreasing curves in gamma frequency strength. We also measured GE and hypothesized to replicate the findings of Khan et al. (2018), who identified a significant nonlinear GE trajectory in the gamma band. We then conducted a PSD analysis to evaluate the relation between whole-brain band-limited PSD and age. Based on the previous EEG literature, we hypothesized PSD to decrease globally with age most strikingly in the theta frequency band. As there have been mixed reports of the relation between PSD and age in higher frequencies, we predicted a significant change with age. Finally, we assessed for any differences in signal-to-noise ratio (SNR) across our sample.

## RESULTS

Following data quality control, a cohort of 324 participants (from an initial group of 425, aged 5.9 to 45.5 years) was included in our analyses (mean age [*SD*]: 18.8 [9.9], 116 females). Resting-state MEG data were epoched into 10-s “trials” and an automated procedure was used to remove trials containing significant artefact or excessive head movement (>7 mm). MEG data were coregistered to individual anatomical MRIs and, for each participant, a single-shell head model constructed. The center of mass of each automated anatomical labeling (AAL) atlas parcel (Tzourio-Mazoyer et al., 2002) was nonlinearly unwarped into subject space, and a beamformer class of spatial filter was used to project MEG data into source space to derive a single signal time course for each AAL parcel. Regional signals were frequency filtered into four classical frequency bands (theta: 4–7 Hz, alpha: 8–14 Hz, beta: 15–30 Hz, gamma: 31–80 Hz) and used to measure PSD and to estimate functional connectivity using the weighted phase lag index (Vinck, Oostenveld, van Wingerden, Battaglia, & Pennartz, 2011). See the Methods section for a schematic illustration of our processing pipeline (Figure 6).

### Grand-Average Functional Connectivity Highlights Spectral Segregation

The weighted phase lag index (wPLI) assumes that a functional connection exists between two regional signals if the phase of one signal consistently leads or lags another over time. Values of wPLI range from 0 to 1, with 0 indicating a completely random/symmetric phase distribution and 1 indicating a highly asymmetric distribution; wPLI was calculated between all node pairs of the AAL atlas, within each frequency band, enabling a whole-brain measure of neural synchronization for each participant.

**Figure 1.**
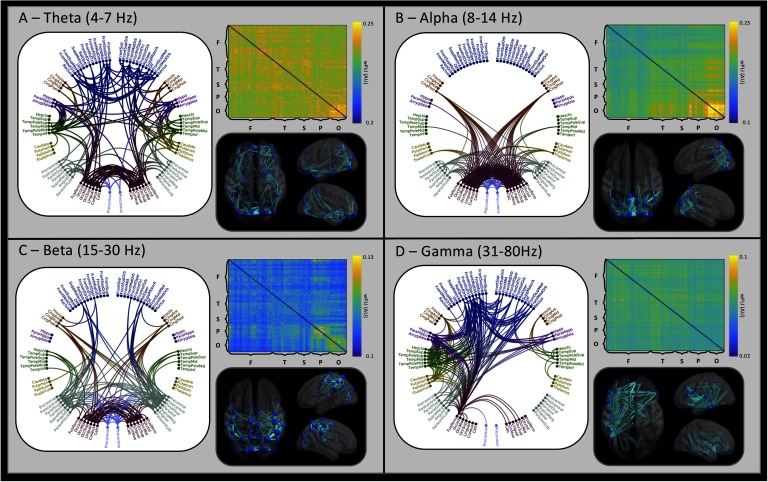
Grand-averaged functional connectivity (wPLI). Within each frequency quadrant, connections falling within the 95th percentile are plotted in the circle plots and glass brains. The circle plots are arranged anatomically such that left hemisphere regions appear on the left of the plot. Regional connections (edges) within the glass brains are scaled within band, with thicker edges indicating a greater connection strength. The spheres indicating individual regions (nodes) are scaled by connectivity strength, with larger spheres indicating greater connectivity values to connected regions, within the 95th percentile of connections. Adjacency matrix labels indicate the lobular segregation of the matrix, in the order of frontal (F), temporal (T), subcortical (S), parietal (P), and occipital (O). Note that adjacency matrix color axes are scaled independently by band.

Grand-averaged adjacency matrices are plotted in Figure 1, accompanied by glass brains and circular connectivity plots showing the top 5% of connections, for each frequency band. Adjacency matrices plot the wPLI value between two brain regions, with greater values indicating a greater functional connection between regions. Matrices are arranged by lobe, with homologous regions plotted in neighboring rows and columns (left then right). The strongest connections within the theta band are broadly distributed, as shown in the circular connectivity plot, whereas the alpha band adjacency matrix is dominated by high occipital phase synchrony, indicated by the bright yellow square. This feature is echoed in the glass brain, with a high concentration of connections within the occipital lobe and a high degree of occipital symmetry in the alpha circle plot. While the beta band glass brain (Figure 1C) also includes many occipital connections, there is a reduced local concentration with many connections entering the parietal lobe, particularly across the motor cortices. The gamma frequency wPLI adjacency matrix is characterized by broadly distributed high connection weights. Plotting the highest 5% of edge weights onto the glass brain highlights a hemispheric asymmetry, with a greater number of connections in the left hemisphere for gamma. The circle plot reveals that many left hemisphere regions connect bilaterally to the frontal lobes and subcortical structures such as the hippocampus.

### Graph Theoretic Measurements Mature With Nonlinear Trajectories

Measurements of node strength and global efficiency were entered into a cross-validation regime, enabling the selection of the best fitting curve, based upon a linear, quadratic or logarithmic model. This technique offers robust curve selection that is not influenced by the complexity or number of coefficients of a curve (see Methods). While other experimenters have chosen model-free techniques to characterize neurodevelopment, such as local regression (Khan et al., 2018), we adopted this fitting regime as our models can be described completely by four or fewer coefficients, facilitating replication. Following completion of the cross-validation, the best fitting curve type was regressed with the full dataset and an empirical significance level, derived from null distributions, was obtained. The resultant *p* values were corrected for multiple comparisons using a false discovery rate correction at a threshold of *q* < 0.05, within each frequency band.

**Figure 2.**
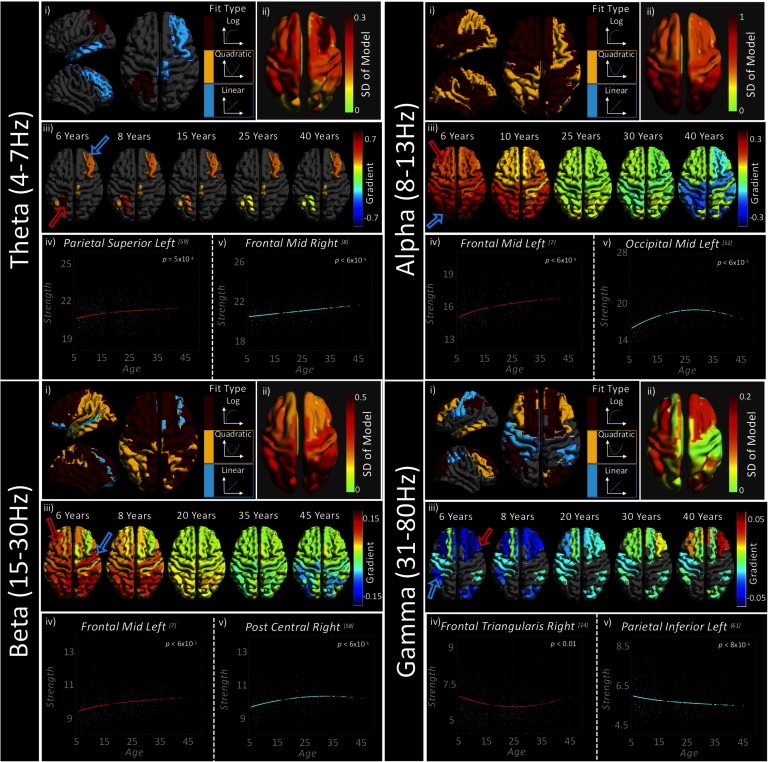
Typical neurodevelopmental trajectories assessed with wPLI-S. Each quadrant (band) contains the following sections: Section (i) plots curve types that were found to be statistically significant on a regional basis. Any regions in gray indicate regions with nonsignificant fits. Section (ii) presents the standard deviation (*SD*) of the model, indicating to what extent the data change over the developmental course. Section (iii) plots axial brain images indicating the gradient between age and wPLI-strength. Hotter colors indicate a steeply increasing gradient (e.g., the upward portion of a quadratic or log fit) and cooler colors indicate a steeply decreasing gradient; note the change in color axes with frequency band. Regions in gray are regions where the best fit was found not to be significant. Arrows on the first brain in (iii) correspond, in color, to the graphs plotted in (iv) and (v), plotting the relationship between age and wPLI-strength, with the colored line indicating the model that best characterizes that regional relationship. See Figures S7 and S8 (Hunt et al., 2019) for videos depicting gradient change from 6 to 45 years of age. Video S7 presents gradients only for significantly characterized curves, and S8 presents all gradients. Table S10 (Hunt et al., 2019) details the best fitting model coefficients for each region and frequency band. We also present a figure focused on subcortical trajectories in the supplementary material, Figure S1 (Hunt et al., 2019).

Figure 2 presents the results of the curve fitting analysis for node strength measurements. The top row of each subfigure (i) first presents the best model type on a regional basis, for statistically significant fits only (*q* < 0.05). The second plot of the top row (ii) presents the extent to which the data change across the life span, calculated as the standard deviation (*SD*) of the best fitting model. The second row (iii) presents the statistically significant curve gradients for exemplar ages. These gradients are also presented as both significance thresholded and unthresholded videos (S7 and S8, respectively; Hunt et al., 2019). The third row presents exemplar graphs (iv and v) for two regions (indicated by colored arrows in [iii]). Note that the color axis of the brain plots has a direct relation to change in node strength, with, for example, a value of 0.7 indicating that for every year of development, node strength increases by 0.7. The theta band is characterized by predominantly linear trajectories, with only left parietal regions fitted with a more complex logarithmic curve. The regions with the greatest change in strength, as indicated by hotter colors in the upper right brain plot, are also those to be significantly characterized by our curves within the theta band. These regions form one half of an asymmetric frontoparietal network. The left graph (red curve) plots an exemplar logarithmic fit, and the right graph (blue curve) plots a linear fit from the right frontal region.

In the alpha band, all regional strength measures were found to be statistically significant, with the logarithmic curve most successfully characterizing the development of node strength. The few alpha band regions that were characterized by a quadratic curve closely match those in theta band that were found to contain significant fits, with an asymmetric frontoparietal distribution. Positive alpha band curve gradients were greatest in the occipital lobes and the highest negative gradients, toward the older participants in our cohort, occur in parietal and sensorimotor cortices, indicating that strength in these regions descends more rapidly with age than those in the occipital regions.

The beta frequency band was significantly characterized by all three curve types, although the majority of regions were best described by a logarithmic model. As observed in the alpha band, occipital regions were found to have the steepest positive developmental gradient. However, within the beta frequency band the negative gradients for quadratic fits were approximately equal across occipito-parietal nodes. The best model for the right somatosensory cortex (postcentral gyrus) is plotted in the right graph (blue); note that the peak of this quadratic fit occurs at approximately 30 years old.

In the theta, alpha, and beta bands, nonlinear models were all positively characterized; node strength first increased with age. In the gamma band the opposite was true, with all linear and nonlinear fits characterized by negative coefficients, indicating reducing strength in childhood. This relation is most apparent in a quadratic form in the frontal regions, which at 6 years old are steeply negative (a 0.05 decrease in strength per year of age) and at 40 years are steeply positive (a 0.05 increase in strength per year).

These patterns are echoed in a detailed analysis of subcortical regions, presented in the Supporting Information (Figure S1; Hunt et al., 2019). The alpha and beta bands were significantly characterized by nonlinear trajectories in all subcortical regions. The theta band was significantly characterized by a positive linear model in the left caudate region. The gamma band was also found to develop linearly, with decreasing strength in the right thalamus and left parahippocampal region.

**Figure 3.**
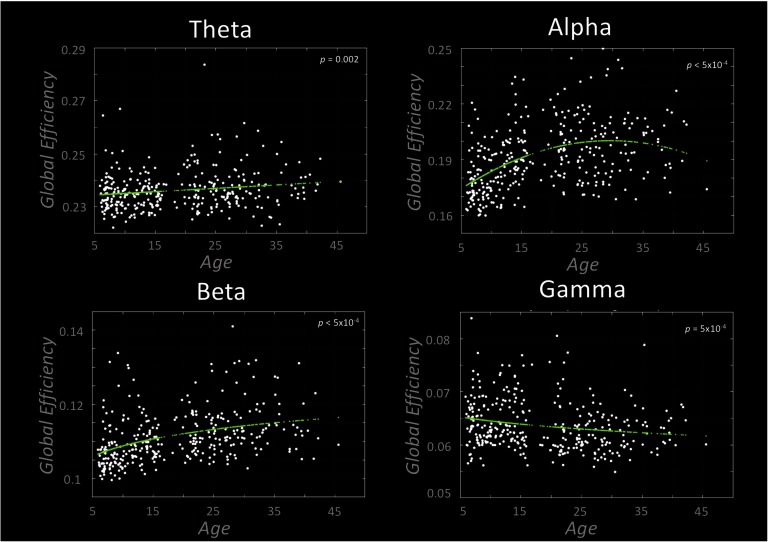
Typical neurodevelopmental trajectories assessed with global efficiency (GE). Each plot presents the statistically significant curve fits following our cross-validation analysis. Theta band GE is significantly characterized by a linearly increasing fit, whereas the alpha and gamma bands were characterized by a quadratic curve. The beta band was significantly characterized by a logarithmic curve.

Figure 3 presents the results of our global efficiency (GE) curve fitting results, where a higher GE score indicates that information is passed more easily throughout the entire network. In all bands, GE development was significant. Theta band GE was found to increase linearly with age. Alpha and gamma band GE was significantly characterized by quadratic curves, the former peaking at 30 years of age. Beta GE was characterized by a logarithmic model.

### Maturation of Spectral Power Is Regionally Specific

**Figure 4.**
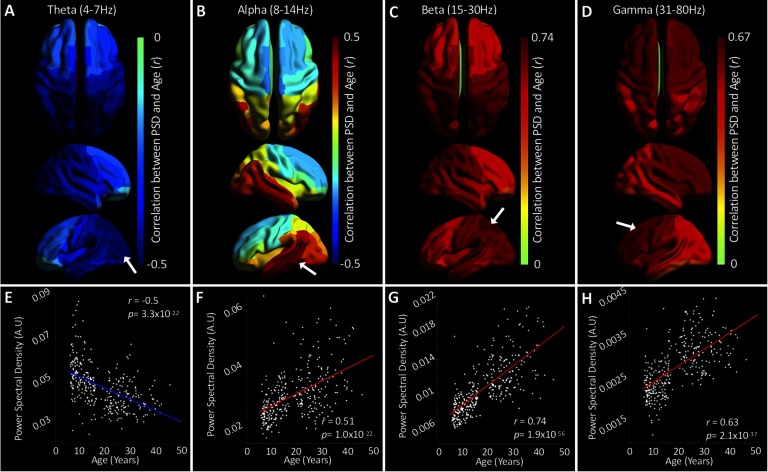
The relationship between age and power spectral density. A–D present regional correlations between PSD and age for each frequency band. Hotter colors indicate a positive correlation and cooler colors indicate the opposite relationship. E–H plot the correlation between PSD and age for exemplar regions (indicated by white arrows in A–D). See Figure S3 (Hunt et al., 2019) for the results of an analysis investigating the extent to which these differences arise because of age-related changes in SNR (A–D) or head motion (E–G).

PSD was calculated for each trial and each subject. Trial-averaged PSD values were then correlated with subject age for each region of the AAL atlas. In keeping with the majority of previous studies (Benninger et al., 1984; Miskovic et al., 2015; Vlahou et al., 2014), we used linear correlational analyses to investigate the relation between age and PSD. Figure 4, A–D, presents regional maps of bivariate Pearson correlation coefficients (*r*) between PSD and age for the four frequency bands. Scatterplots for exemplar regions (indicated by white arrows) for each band are presented in Figure 4, E–H.

Theta band PSD was negatively correlated with age in all regions, with maximally negative correlations occurring in the occipital lobe. Alpha band PSD comprises both positive and negative correlations with age, with negative correlations confined to the superior frontal and anterior parietal regions. Beta band PSD was positively correlated with age in all regions, with maximal correlation occurring on the superior border between occipital and parietal lobes. Finally, gamma band PSD estimates were also found to be positively correlated with age in all regions, albeit to a marginally lower extent than those in the beta band.

To further investigate our PSD results, we performed supplementary analyses. The first, presented in Figure S2 (Hunt et al., 2019), investigated the presence of heteroskedasticity of PSD values across our age range; we found little evidence for systematic effects aside from the theta band, where the variance in all regions was found to be greater in younger subjects. Our second analysis demonstrated quantitatively that PSD effects were not driven by SNR changes across our cohort (Figure S3; Hunt et al., 2019).

### Signal-to-Noise Ratio Is Related to Age

**Figure 5.**
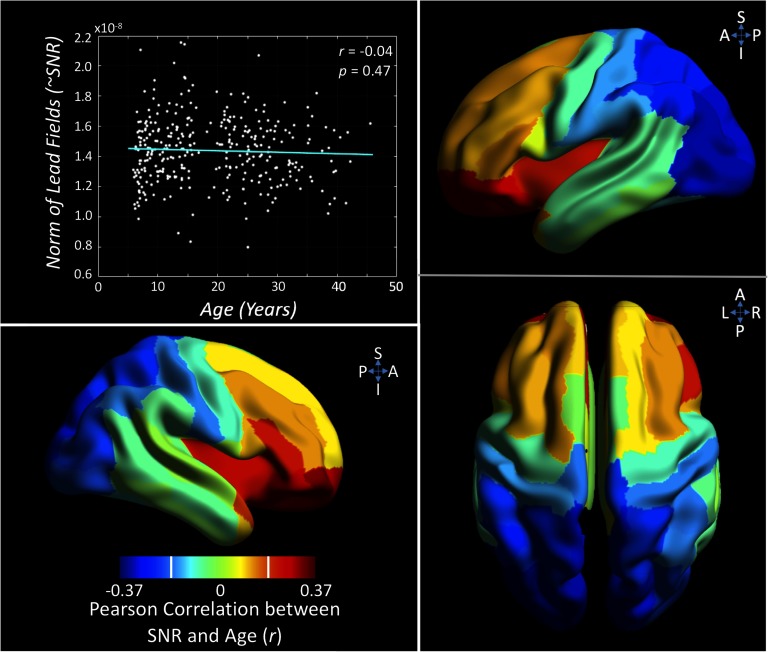
Assessing the influence of age on signal-to-noise ratio. The upper left graph plots the correlation between a proxy for global signal-to-noise ratio (SNR; Frobenius norm of the lead fields) and age (nonsignificant, *p* > 0.05). The brain plots expand on this relation by plotting the Pearson correlation between SNR and age on a regional basis. The white vertical bars on color scale indicate threshold for significant correlation (*r* = ±0.19). See Figure S5A (Hunt et al., 2019) for a greater exploration of this effect.

Our sample spans an age range of almost 40 years, containing data from a diverse set of head/brain sizes. As the MEG signal decreases rapidly with distance, it is important to assess whether the age of the participant significantly impacts our ability to accurately measure neuromagnetic signal. Given the known head size and the location of the sensors with respect to the brain, we modeled the expected signal-to-noise ratio individually for every subject. We found no significant effect of age on the average SNR across the head (*r* = −0.04, *p* = 0.47). Figure 5 also plots the regional correlation values between SNR and age; here a negative correlation implies greater SNR for younger subjects, and a positive correlation the opposite. Note that the threshold for a significant regional correlation is *r* ≥ 0.19 (t > 3.5, p_corr_ < 0.05). This spatial analysis revealed younger subjects to have significantly greater SNR in occipital regions and reduced SNR in inferior frontal regions. Figure S5A (Hunt et al., 2019) presents a further exploration of this, plotting SNR against age for a frontal and occipital region.

## DISCUSSION

Typical neurodevelopment is a highly complex process with many different cellular changes occurring over many timescales (Tau & Peterson, 2010). Characterization of these trajectories is key if we are to understand the developmental origins of complex human behaviors as well as neurological and cognitive pathology. In the present study, we used magnetoencephalographic measurements of power spectral density (PSD) and functional connectivity (FC) to characterize developmental trajectories as measured by this powerful technique. Our sample is the largest currently available, placing us in a unique position to conduct a well-powered foundational study. Our results replicate and extend the previously reported negative relationship between theta band PSD and age, and add to the weight of evidence that higher frequency bands (beta and gamma) exhibit globally reversed correlation coefficients compared with slow-wave oscillatory power. Our calculation of weighted phase lag index (wPLI) revealed spatially different topologies for the strongest connections within each frequency band, with alpha wPLI, for example, characteristically marked by dense occipito-occipito connections. Moreover, node strength, based upon measurement of wPLI, was found to exhibit complex relations with age in a regional and frequency-specific manner, a pattern mirrored in measurements of global efficiency.

### Power Spectral Density: Replication and Novelty

The finding of a negative relationship between theta band PSD and age has been reported widely (Miskovic et al., 2015; Rodriguez-Martinez et al., 2015; Smit et al., 2012; Whitford et al., 2007). Three studies, to the authors’ knowledge, have reported age-related increases in PSD in higher frequencies, and our results buttress these findings (Benninger et al., 1984; Clarke et al., 2001; Perone et al., 2018). We report, for the first time, frontal decreases and occipital, parietal, and temporal increases in alpha band PSD with age (Figure 4B). Whitford et al. (2007) argued the physiological basis of PSD is linked to cortical volume, as the team found similar relations between age and PSD and age and cortical volume. While our current results preclude direct comment on this claim, our findings revealing PSD to be both positively and negatively correlated with age in the alpha band suggests a more complex relationship. Whitford et al. used voxel-based morphometry to derive measures of cortical volume. Although this technique has been used extensively, a more detailed approach is to measure different properties of the cortex as opposed to volume, such as cortical thickness, local gyrification, and surface area, and to make inferences on microstructural properties such as myelination (Geades et al., 2016). These measures have different developmental trajectories (Winkler et al., 2010) and relate nontrivially to one another. It would therefore be pertinent for future studies to investigate the relationship between these more detailed measurements of cortical structure and PSD.

### Grand-Average wPLI: Insights from Gamma Band Asymmetry

Figure 1 plots the grand-averaged wPLI, collapsing across age groups. Taking the strongest 95% of connections, striking symmetry is seen in the theta, alpha, and beta bands. This is in contrast to the profound asymmetry seen in the gamma band. This asymmetry is biased toward the left hemisphere with many within-hemisphere connections. Gamma frequency oscillations are produced in superficial cortical layers (Buffalo, Fries, Landman, Buschman, & Desimone, 2011) and are therefore considered to be primarily associated with local communication. The left hemisphere has been found to be biased toward within-hemisphere connections (Gotts et al., 2013), whereas the right hemisphere shows no such bias. This may underlie the asymmetry in the group-averaged gamma band plots.

### wPLI-S and GE: Windows to Cognitive and Sensory Development

Our analysis of wPLI-strength (wPLI-S) revealed highly heterogeneous developmental trajectories both within and between frequency bands. The major trends across bands were echoed in GE analysis. It is noteworthy that quadratic and asymptotic/logarithmic models suggest distinct underlying processes. Quadratic model fits indicate processes that are continually evolving across the life span (or sample age range). A quadratic form may arise either from a single physiological phenomenon with a quadratic trajectory, or from two nontemporally coincident processes, the second of which occurs at the peak or trough of the quadratic form. In contrast, a logarithmic model suggests an early period of rapid change that gradually plateaus.

Theta band wPLI-S was characterized primarily by linear relations with age, albeit in a small number of regions. Theta is understood to be a frequency band associated with long-range cognitive communication in the brain; the linear relations seen may reflect the steady increase in cognitive abilities across age, even into mid-adulthood. This process would involve increasing the capacity for information transfer in the global network, explaining the steady increase in GE across our cohort. We also found two regions in the left parietal cortex to be characterized by logarithmically increasing wPLI-S with age. This brain region is particularly involved in writing (Menon & Desmond, 2001), and this more rapid early increase may index the acquisition and refinement of this skill in our younger participants.

Generators of alpha frequency oscillations have been identified across the cortical laminae (Bollimunta, Mo, Schroeder, & Ding, 2011; Dougherty, Cox, Ninomiya, Leopold, & Maier, 2017), suggesting that they may modulate both local and long-range connectivity (Uhlhaas, Haenschel, Nikolić, & Singer, 2008). Alpha band oscillations have been linked closely with cortical inhibition (Pfurtscheller & Lopes da Silva, 1999), which is thought to be a key proponent of healthy brain function (Klimesch, 2012), as well as memory (Freunberger, Fellinger, Sauseng, Gruber, & Klimesch, 2009; Jensen, Gelfand, Kounios, & Lisman, 2002; J. Sato et al., 2018), an essential cognitive skill. Several reports indicate that cognitive processes requiring cortical inhibition, such as cognitive control (Houghton & Tipper, 1996), continue to develop in adolescence and adulthood (Adleman et al., 2002; Rubia et al., 2006), as do mnemonic skills (Ornstein & Light, 2010). Here, we found alpha band wPLI-S to be characterized predominantly by logarithmic curves in the frontal lobes, and an approximately even distribution of quadratic and logarithmic curves in other regions. Given the protracted development of the complex cognitive activities associated with alpha oscillations, our nonlinear characterization of alpha band wPLI-S extends the existing literature pertaining to this ubiquitous frequency band; its widespread effects underscore the importance of cortical inhibitory and memory functions across all regions in the brain. The rapid frontal increases in childhood are consistent with the emergence of increasing ability in these executive functions, and the quadratic curves are consistent with some later decline, particularly in memory. This potential decline is supported by the GE results, which suggest that the peak efficiency of the network is at approximately 30 years old. Further, GE is most sensitive to short-range connections, meaning that the regions with logarithmic wPLI-S may be preferentially involved in long-range subnetworks.

Similar to the alpha band results, our analysis revealed beta band wPLI-S to be predominantly characterized by nonlinear curves. Prior studies have found beta band graphs to be closely linked to brain structure (Hunt et al., 2016; Tewarie et al., 2014), which is known to develop nonlinearly (Paus et al., 2001; Shafee et al., 2015). Invasive electrophysiological studies have identified beta band oscillations to be generated in deeper cortical layers (Sun & Dan, 2009), principally implicating this frequency in long-range communication (Brookes et al., 2011), and in motor control (Weinrich et al., 2017). We observe high symmetry between motor cortices, both being fitted with logarithmically increasing curves with similar curve gradients. While the majority of motor maturation occurs before the youngest members of our cohort, motor refinement continues through adolescence and into early adulthood (Smits-Engelsman & Wilson, 2013), which may explain the logarithmic trajectories of the pre- and postcentral gyri. We also observed the highest degree of hemispheric symmetry within beta band curve gradients—potentially implicating a connectivity preference for homologous connections.

In both the alpha and the beta band, the frontal lobes are predominantly characterized by logarithmic curves, whereas the parietal, temporal, and occipital lobes include a high number of quadratic fits. Our measurement of wPLI-S is indicative of overall connection strength to other brain regions. As such, it is tempting to argue that the rapid increase in frontal lobe connectivity is temporally coincident with both the selective strengthening of increasingly important functional connections, and the physical pruning of redundant connections— generating a net increase in wPLI-S that plateaus post-adolescence (Uhlhaas, 2011). This theory is supported by the GE results, as the pruning of redundant connections would lead to increased efficiency in the global network. The regions that tended toward a quadratic trajectory in alpha and beta band wPLI-S are similar to the network identified by Douaud et al. (2014), encompassing transmodal regions within the parietal, occipital, and temporal lobes. The group found this network to be late to mature in youth and early to decline in old age, and also demonstrated these regions to a show heightened vulnerability to disorders occurring early (early onset psychosis) and late (Alzheimer’s disease) in the life span. The quadratic curves we found may be the early harbingers of later declines in function in these brain areas.

We found gamma frequency PSD to increase globally with age in addition to negative nonlinear trajectories with connectivity strength. We interpret this as a concomitant increase in local processing (i.e., within the cortical parcel) and decrease in long-range integration. These results replicate, to some extent, the results recently presented by Brookes et al. (2018), who, in a smaller study, found whole-brain strength (regional strength summed across all parcels) to decrease nonlinearly in the gamma frequency band. In the current study, regions exhibiting the greatest developmental increase in gamma power (Figure 4D) were those bilateral superior-frontal parcels that also exhibit nonlinearly decreasing gamma strength trajectories (Figure 2). This is consistent with the prolonged maturation of the frontal lobes and is associated with the greatest age-related changes in cognitive functions. These changes are in contrast to fewer frontal effects seen in the other frequency bands, underscoring the possible linkage between the frontal regions and the executive functions they support, within the gamma frequency. Alternatively, the more general effects seen in the lower frequency bands, particularly alpha, would reinforce the protracted development of widespread networks that are necessary for human cognition.

We hypothesized GE in the gamma band to take a quadratic (inverted U) form, given the results of Khan et al. (2018). We did find gamma had a quadratic form, but with an opposite sign to the findings by Kahn et al. (a U rather than an inverted U). While our approach is very similar to their study, we chose to use a phase-based metric of connectivity (wPLI), whereas Kahn and colleagues used amplitude envelope connectivity. While wPLI and AEC are undoubtedly related, they measure neuronal processes on different timescales. AEC-based analyses assess connectivity at a temporal resolution of ∼1 Hz (the resolution of the envelope of amplitude changes), whereas phase-based techniques remain in the millisecond range. Khan and colleagues used a fine-grained parcellation including more than 400 cortical regions, whereas we used the more commonly selected AAL atlas (Tzourio-Mazoyer et al., 2002) consisting of 90 parcels, including cortical and subcortical regions. Further, our age range is larger and more evenly distributed than that used by Kahn et al. (7 to 29 years, with the greatest density of participants >20 years), which may mean that the early life trajectory was poorly characterized in their curve fitting. Therefore, we believe this discrepancy may be accounted for by the differing temporal and spatial scales at which the graph metrics are computed and the different characteristics of our cohorts.

### Future Directions and Limitations

The models used in the current study, and the methods used to create them, are highly generalizable across laboratories and imaging modalities. As such, we encourage future studies to incorporate these models into their research and assess how their data converges or deviates from these trajectory patterns. Similarly, we encourage those with data from clinical cohorts to assess how their findings diverge from this general model of typical development. While we derived this model from MEG data, it would be interesting to assess the overlap between MEG and EEG as well as MEG and fMRI.

The cohort used in this study was limited in age span by the fundamental physics of traditional MEG systems, where participants younger than approximately 5 years of age will have heads too small, and subsequently would be too far from sensors, for reasonable SNR in MEG. To perform MEG in younger subjects, several targeted systems exist that are specifically designed to have smaller dewars such that the sensors are closer to the heads of young children (e.g., He et al., 2018) or for infants (e.g., Gaetz et al., 2015). There have also been exciting developments in next-generation MEG sensors, in particular optically pumped magnetometers (OPMs; Boto et al., 2016; Boto et al., 2018), and necessary magnetic field nulling technology (Holmes et al., 2018). These new sensors can be placed directly onto the scalp, meaning an increase in SNR and negation of head movement–related artefacts. With continued development and optimization, these sensors will provide new possibilities for MEG recordings in neonates and young children.

A further limitation is that our sample was unevenly distributed between the sexes (see Figure S9 in the Supporting Information; Hunt et al., 2019), meaning that it is possible our results are more applicable to males than females. To assess this empirically, we performed 100 additional analyses with subsampling of males to match females, totaling 540 million permutations (see the Supporting Information, section 1.4; Hunt et al., 2019) and found alpha, beta, and gamma band wPLI-S curve types to be stable even when sexes were matched. Theta band wPLI-S was less stable, potentially implicating that sexual dimorphisms mainly localize to this band. This would also explain why there were few significant fits in the primary analysis of theta band wPLI-S. Assessment of sex differences in the theta band would be an exciting direction for future research.

In conclusion, we have performed extensive investigation of typical human neurodevelopment using a large developmental MEG database. We found PSD to closely relate to age in a frequency- and region-dependent manner. wPLI-S was significantly characterized as linearly, quadratically, and logarithmically changing across development. Alpha and beta frequency bands best characterized the relationship between wPLI-S and age, with the greatest number of significantly fitted regions. Gamma band wPLI-S was found to exhibit opposite relations to other bands, suggesting a drop in long-range connectivity with increasing age. GE was significantly associated with age in all frequency bands, also revealing spectrally distinct linear and nonlinear trajectories, largely consistent with wPLI-S. We have shown PSD and wPLI-S to be highly sensitive to the fundamental functional architecture of the developing brain. Our foundational study provides a concrete footing from which future studies can detail links with specific cognitive functions, and clinical research may begin to decisively map divergent pathological trajectories in neurodevelopmental disorders.

## MATERIALS AND METHODS

### Human Subjects

A retrospective database was assembled consisting of 425 typically developing research participants from The Hospital for Sick Children (SickKids), scanned between August 2010 and June 2017. Participants were recruited to a number of different projects led by MJT and BTD. To be included in the present analysis, participants were required to be free of MEG or MRI contraindications, neurological disorders, and any developmental or psychiatric disorders, and to have completed a 5-min resting-state acquisition with a static fixation cross. Following exclusion based on task completion and preprocessing of data (including excessive head movement [>7 mm]), a final cohort of 324 participants (mean age: 18.8, standard deviation: 9.9, 116 females, range: 5.9 to 45.5 years; for sex distribution see Figure S9, Hunt et al., 2019) were included in the main analyses. All studies received ethical approval from The Hospital for Sick Children Research Ethics Board.

### MEG and MRI Data Acquisition

Five minutes of resting-state MEG data were acquired in supine position using a CTF 151-channel system (CTF-MISL, Coquitlam, Canada) positioned within a magnetically shielded room (MSR). Participants were instructed to fixate on a centrally positioned gray cross (+) within a circle on a black background, back projected onto a screen inside the MSR. Data were acquired at a sampling frequency of 600 Hz operating in third-order synthetic gradiometry configuration. Prior to acquisition, participants were fitted with three head position indicator coils, located at the nasion and left and right preauricular points. These coils were tracked continuously, enabling a moment-to-moment measurement of head motion. The location of these coils was recorded, and MRI-visible markers positioned at these locations to coregister between MEG and MRI data.

An anatomical T1-weighted image was also acquired for all participants using a 3T Magnetom Tim Trio (Siemens AG, Erlangen, Germany) MRI system using a 12-channel head coil running an MPRAGE pulse sequence (TR = 2,300 ms; TE = 2.9 ms; flip angle = 9°; field of view = 240 × 256 × 192 mm; slice thickness 1 mm^3^).

### Preprocessing

**Figure 6.**
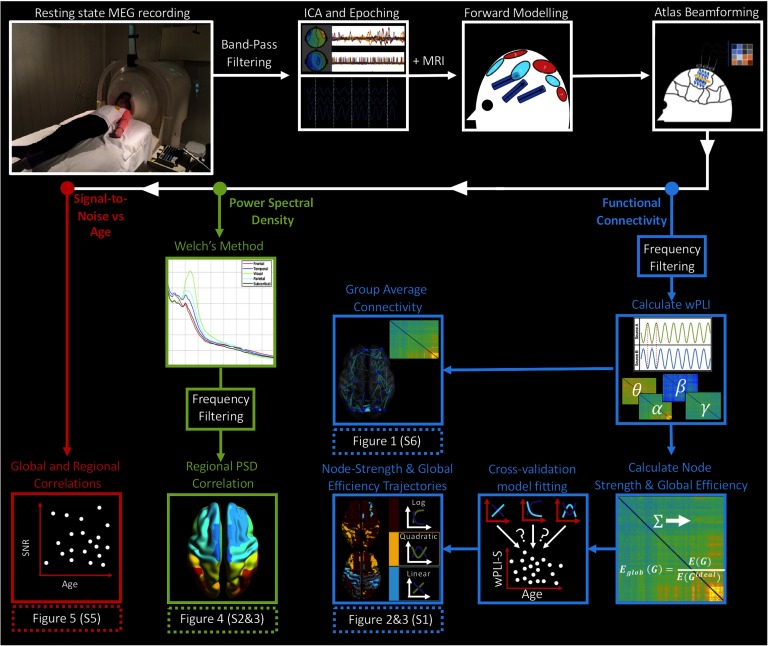
Processing pipeline schematic. MEG data were first bandpass filtered, and independent component analysis (ICA) was used to de-noise the data. Following this, data were epoched into 10-s “trials” (for processing efficiency) and beamformed to parcels of the AAL atlas. Functional connectivity (blue): Data were frequency filtered into bands and functional connectivity calculated using the weighted phase lag index (wPLI). wPLI-S and GE were entered into a cross-validation curve/model fitting algorithm, which fit either linear, quadratic, or logarithmic curves to data, quantifying changing graph properties with age. Power spectral density (green): Using atlas-beamformed data, we used Welch’s method to calculate PSD. These values were binned into frequency bands and Pearson’s correlation was used to infer relations between PSD and age. Signal-to-noise (red): Using the lead fields generated to solve the forward problem, we investigated whether any relation between SNR and age existed, using both global and regional SNR/lead field values. Note that figures in brackets appear in the Supporting Information section (Hunt et al., 2019).

Figure 6 presents a schematic of our processing pipeline.

First, data were bandpass filtered between 1 and 150 Hz, and notch filters were applied at 60 and 120 Hz. Second, an independent component analysis (ICA) was performed on MEG data (Muthukumaraswamy, 2013). A single experimenter examined components and those deemed arising from eye blinks or cardiac muscles were removed from the data. Next, an automated data rejection algorithm was used to detect and mark segments of data containing excessive head movement (>7 mm) or artefact. The remaining clean data were epoched into as many 10-s “trials” as available. Note that these trials are arbitrary divisions of a continuous resting-state recording and are epoched for computational reasons. Participants with greater than five trials remaining following this procedure were taken forward for primary analyses. The mean number of trials per participant was 24.5.

### Forward Model and Beamforming

A linearly constrained minimum variance (LCMV; Van Veen, van Drongelen, Yuchtman, & Suzuki, 1997) spatial filter, implemented in Field Trip (Oostenveld, Fries, Maris, & Schoffelen, 2011), was used to enter source space. The forward model was based upon a dipole approximation (Sarvas, 1987) and a realistically shaped single-shell approximation (Nolte, 2003). Dipole orientation was determined using the maximum eigenvector and fixed across trials. Covariance was calculated within a 1–150 Hz covariance window spanning the entire experiment and regularized using the Tikhonov method with a regularization parameter equal to 5% of the maximum eigenvalue of the unregularized matrix. The LCMV output was normalized by estimated noise, the result being termed the neural activity index (NAI), to avoid biasing measurements toward the center of the head. Subject-level data were transformed onto the 90-parcel AAL atlas (Tzourio-Mazoyer et al., 2002) and a beamformer spatial filter was used to derive a single signal from each region at the region’s center of mass.

### Assessing for Age-Related Confounds

Given that dipolar magnetic field, the source of the MEG signal, falls with a $\frac{\text{1}}{{\text{r}}^{\text{2}}}$ relation, head size may represent a significant confound in MEG studies of development, as smaller heads may be accompanied by reduced signal-to-noise ratio. To ensure that our results were not biased by head size, an assessment of SNR was performed using each subject’s lead fields. The forward field (forward model) models the field strength at the sensor level arising from a dipole of unit strength at a given voxel (Robinson & Rose, 1993). Derivation of forward fields takes account of the subject’s head position relative to MEG sensors. Measurement of the average forward fields for each subject provides a measure of SNR per subject. We calculated forward fields from the center of mass of each AAL region for each subject. We then took the root mean square over orientations and, for the overall relation, the Frobenius norm over channels and regions, and the mean over channels for individual region correlations. These results were plotted against age, and bivariate Pearson’s correlation was used to assess for a statistically significant relationship.

We also assessed for age-related differences in the number of trials per subject. As would be expected, data from younger subjects were prone to greater head movement and number of artefacts, therefore more trials from younger subjects were removed during the artefact rejection procedure. To counteract this, we calculated the average number of trials for participants less than 11 years old (21 trials) and randomly removed trials from the data of older subjects with a greater number of trials than this threshold.

Our Supporting Information (Hunt et al., 2019) presents further analyses assessing for age-related confounds. Figure S5A presents an expanded analysis of the SNR plot from Figure 5. Here we plot the SNR from exemplar regions, in the frontal and occipital lobes, against participant age. Figure S5B assesses the influence of the trial balancing procedure described above. Plotting the number of trials against age both before and after the balancing reveals the removal of significant relations between age and the amount of data entered into analyses.

### Power Spectral Density (PSD) Calculation

Trial-wise regional time courses, generated by the beamformer spatial filter, were mean-centered. PSD was then estimated using Welch’s method (‘pwelch’ function in Matlab [MathWorks, Natick, USA]). The resultant values were trial-averaged and portioned into four frequency bands of interest (theta: 4–7 Hz, alpha: 8–14 Hz, beta: 15–30 Hz, gamma: 31–80 Hz) and for each, the mean in the frequency domain derived. To characterize the relationship between age and PSD, bivariate Pearson correlations were computed between participant ages and PSD estimates for each frequency band and region. Regional PSD correlations were plotted using Brain Net Viewer (Xia, Wang, & He, 2013).

### Calculating Weighted Phase Lag Index Strength and Global Efficiency

The weighted phase lag index (wPLI) estimates the asymmetry, around zero, of the phase difference between two signals weighted by the magnitude of the imaginary cross-spectrum. It is assumed that a functional connection exists between two regional signals if one signal consistently leads or lags another over time. wPLI values range from 0 to 1, with 0 indicating a completely random/symmetric phase distribution to 1, indicating a highly asymmetric distribution. This technique is advantageous as compared with the original phase lag index (Stam, Nolte, & Daffertshofer, 2007) as it has been shown to be less sensitive to volume conduction and noise sources more generally (Vinck et al., 2011) and has been used successfully in many EEG and MEG studies. Trial-wise, frequency filtered, regional time courses were entered into an in-house-coded wPLI routine, exactly following the procedure described in the original publication (Vinck et al., 2011). Time courses were mean-centered before using the Hilbert transform to extract the instantaneous phase of the signal for each region-pair, trial, frequency band, and subject. Trial-wise wPLI values were mean-averaged to generate a single phase synchronization value per region, frequency band, and subject. This process generated an adjacency matrix for each subject and frequency band, characterizing whole-brain phase synchronization. To generate the grand-average adjacency matrices in Figure 1, a mean-average across all participants was derived. Glass brains were plotted using Brain Net Viewer software (Xia et al., 2013). To calculate wPLI-strength (wPLI-S), the weights for each regional AAL node were summed in each frequency band, generating an [AAL nodes (90) × frequency bands (4)] array of values for each subject; this is equivalent to summing across a single dimension of the adjacency matrix. To calculate global efficiency (GE), we used the corresponding function (‘charpath’) in Brain Connectivity Toolbox (Rubinov & Sporns, 2010). This function requires a distance matrix, which was created by taking the inverse of the individual wPLI adjacency matrices and using the ‘distance_wei’ function implemented in Brain Connectivity Toolbox.

### Curve Fitting Algorithm

First, we constructed models for the linear (1), quadratic (2), and logarithmic (3) curves as follows:

$$y=a_{1}x+a_{2},$$ (1)

$$y=b_{1}x^{2}+b_{2}x+b_{3},$$ (2)

$$y=c_{1}\log_{10}\left(c_{2}x+c_{3}\right)+c_{4},$$ (3)

where *x* represents subject age, *a*_1–2_, *b*_1–3_, and *c*_1–4_ represent coefficients, and *y* represents our wPLI-S or GE measurements for all participants. Then, for each frequency, and for wPLI-S region, we performed a cross-validation routine. First, we randomly split subject-level data into two equal groups, termed Fit and Test. Second, all three models were fitted to the Fit group. Third, these same models were then fitted to the Test group and the residual difference, derived as the absolute difference between the model and the data, was recorded. This process was repeated 1,000 times, with 1,000 different random group allocations. Following completion of the iterations, the best fitting model was chosen as that with the lowest median residual. By taking the median residual across iterations, we protect against overfitting and ensure that the model selection is not biased toward more complex model forms. Note that the coefficients for all best fitting models are detailed in Table S10 (Hunt et al., 2019).

### Determining Statistical Significance

To determine whether the curve characterizing the relationship between wPLI-S/GE and age was significantly better than chance, we derived an empirical null distribution. This was based on the reasoning that if age had no effect on the graph metric, then randomly shuffling the ages would have no effect on the estimated curve *F* statistic. First, the best fitting model was fit to the entire dataset for a region and frequency band and the *F* statistic derived. Next, the best fitting model was fit to data with the ages randomly shuffled and a null *F* statistic calculated. This process was repeated 15,000 times to generate a distribution of null *F* statistics. A significance value was then derived as the proportion of null *F* statistics less than that for the nonshuffled data. To control for multiple comparisons across regions, strength measurements were thresholded using a false discovery rate, implemented using the Benjamini-Hochberg procedure, at *q* < 0.05.

### Calculating Curve Gradients

To visualize the trajectories more clearly, we calculated the gradients (∇) of each curve type for each year of age (*x*_*n*_),

$$\nabla_{linear}=a_{1},$$ (4)

$$\nabla_{quadratic}=2(b_{1}x_{n})+b_{2},$$ (5)

$$\nabla_{\log}=\frac{c_{1}c_{2}}{\left(c_{2}x_{n}\right)\text{ln}\left(10\right)+c_{3}}.$$ (6)

We used the calculated gradients to visualize maturation, both on static brains and as videos (S7 and S8; Hunt et al., 2019), using Brain Net Viewer (Xia et al., 2013), where each frame (1/s) presents the gradient values for each region in that year of age.

## ACKNOWLEDGMENTS

We wish to acknowledge the following individuals who acquired the data used in this publication: Rachel Leung, Vanessa Vogan, Sarah Mossad, Julie Sato, Veronica Yuk, MyLoi Huynh, and Julie Lu. We also wish to thank the participants who kindly donated their time to multiple studies at The Hospital for Sick Children. Finally, we wish to acknowledge Professor Krish Singh of Cardiff University Brain Research Imaging Centre (CUBRIC), who kindly shared his code for plotting the circular connectivity graphs.

## AUTHOR CONTRIBUTIONS

Benjamin Alexander Edward Hunt: Conceptualization; Data curation; Formal analysis; Investigation; Methodology; Project administration; Software; Visualization; Writing – original draft; Writing – review & editing. Simeon M. Wong: Data curation; Formal analysis; Methodology; Software; Writing – review & editing. Marlee M. Vandewouw: Methodology; Software; Visualization; Writing – review & editing. Matthew J. Brookes: Conceptualization; Methodology; Supervision; Writing – review & editing. Benjamin T. Dunkley: Conceptualization; Methodology; Supervision; Writing – review & editing. Margot J. Taylor: Conceptualization; Funding acquisition; Methodology; Supervision; Writing – original draft; Writing – review & editing.

## FUNDING INFORMATION

Margot J. Taylor, Canadian Institutes of Health Research (http://dx.doi.org/10.13039/501100000024), Award ID: MOP-106582. Margot J. Taylor, Canadian Institutes of Health Research (http://dx.doi.org/10.13039/501100000024), Award ID: MOP-119541. Margot J. Taylor, Canadian Institutes of Health Research (http://dx.doi.org/10.13039/501100000024), Award ID: MOP-137115. Margot J. Taylor, Canadian Institutes of Health Research, Award ID: MOP-142379. Benjamin Alexander Edward Hunt, Hospital for Sick Children (http://dx.doi.org/10.13039/501100006126), Award ID: Restracomp. Benjamin Alexander Edward Hunt, Hospital for Sick Children (http://dx.doi.org/10.13039/501100006126), Award ID: The Centre for Brain and Mental Health Abe Bresver Fellowship.

## TECHNICAL TERMS

Functional connectivity: Nondirectional statistical relations between signals measured from disparate brain regions thought to indicate physiological communication.
Neural oscillations: Rhythmic changes in magnetic field produced by populations of neurons; a key mechanism of interregional communication in the brain.
Power: The amplitude squared.
Power spectral density: A measure of a signal’s power content at specific oscillatory frequencies.
Resting state: Measuring changes in brain activity when the subject is at rest, typically passively viewing a fixation cross.
Amplitude: Change of a neural oscillation from its mean value.
Amplitude envelope connectivity: Functional connectivity measured by assessing correlations over time between the amplitudes of electrophysiological signals from two brain regions.
Phase synchrony: A term to describe a shared timing of the peaks and troughs of oscillations/brain waves between two regions.
Beamformer: A class of spatial filter used to infer the region of the brain responsible for magnetic field changes measured by MEG sensors.
Adjacency matrix: A way of presenting brain connectivity data. Each matrix element corresponds to the connection strength between two brain regions.
Edge weights: A graph theory term for the connection strength between nodes/regions of a graph/network.
Gradient: The rate of change or “steepness” of a curve.
Brain parcellation: A division of the brain based on structural or functional features into a number of discrete regions or parcels.
