# Supplementary material for: Spatial and spectral trajectories in typical neurodevelopment from childhood to middle age
Source: Netw Neurosci. 2019 Mar 1;3(2):497–520. doi: 10.1162/netn_a_00077 (PMC6444935; doi:10.1162/netn_a_00077)
Supplement: Supplementary file 1 [file netn-03-497-s001.pdf]

Hunt, B. A. E., Wong, S. M., Vandewouw, M. M., Brookes, M. J., Dunkley, B. T., & Taylor, M. J. (2019). Supporting information for "Spatial and spectral trajectories in typical neurodevelopment from childhood to middle age." *Network Neuroscience*, *3*(2), 497–520. https://doi.org/10.1162/netn_a_00077

Spatial and spectral trajectories in typical neurodevelopment from childhood to middle age: Supplementary Material

Our supplementary material is split into three sections. The first details the methods and results of further analyses while the second presents further visualisation on results presented in the main manuscript. The third section includes tables detailing the coefficients for the model fits presented for our wPLI-S cross-validation analysis, with corresponding significance values.

1. Supplementary Analyses

1.1 Subcortical Analysis of wPLI-S

Our preferred method for plotting the results of our wPLI-S curve fitting analysis involves colouring parcels on the surface of a template brain. This plotting, however, precludes presentation of results from subcortical parcels. In total, 14 regions were not plotted in Figure 2, seven per hemisphere. The curve fitting results for these regions are shown below in Figure S1.

DOI: 10.1162/netn_a_00077

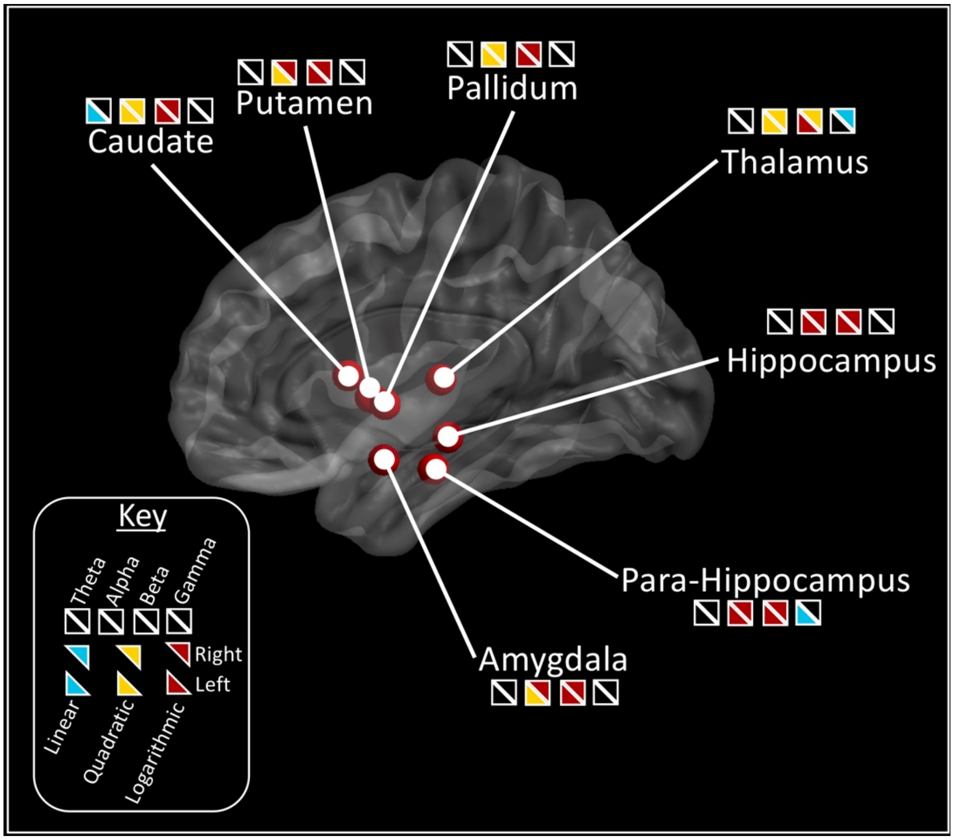

Figure S1. ***Overview of Subcortical Results from wPLI-S Curve Fitting.*** *Above, we present the results of the 14 (seven per hemisphere) nodes that were not presented in the main manuscript. Each node is associated with four boxes, made of eight triangles. The left-most box represents the theta band, with the frequency band increasing when moving right (the right most box represents the gamma band). The triangles that make each box represent the left or right homologue of that region (the side of the right-angle of the triangle indicates which hemisphere). Finally, the colour of each triangle represents which fit type significantly characterised the trajectory from that region, with blue indicating a linear fit, yellow for a quadratic relationship between wPLI-S and age, and red for a logarithmic trajectory. Unfilled triangles indicate that the region was not significantly characterised by any of our curves. Note that nodes plotted on the glass brain represent the centre of mass of that parcel.*

1.2 Investigating Heteroscedasticity of Power Spectral Density Across the Cohort

On closer inspection, the scatter plots presented in Figure 4 suggest that the variance of PSD across age is not equal, but is heteroscedastic across the cohort. To investigate this quantitatively, we split our data into a younger group, aged less than 18 years, and an older group (aged >18 years). For each frequency band and region, we performed a series of Levene's tests for equality of variance. The resulting alpha values for these tests are plotted in Figure S2.

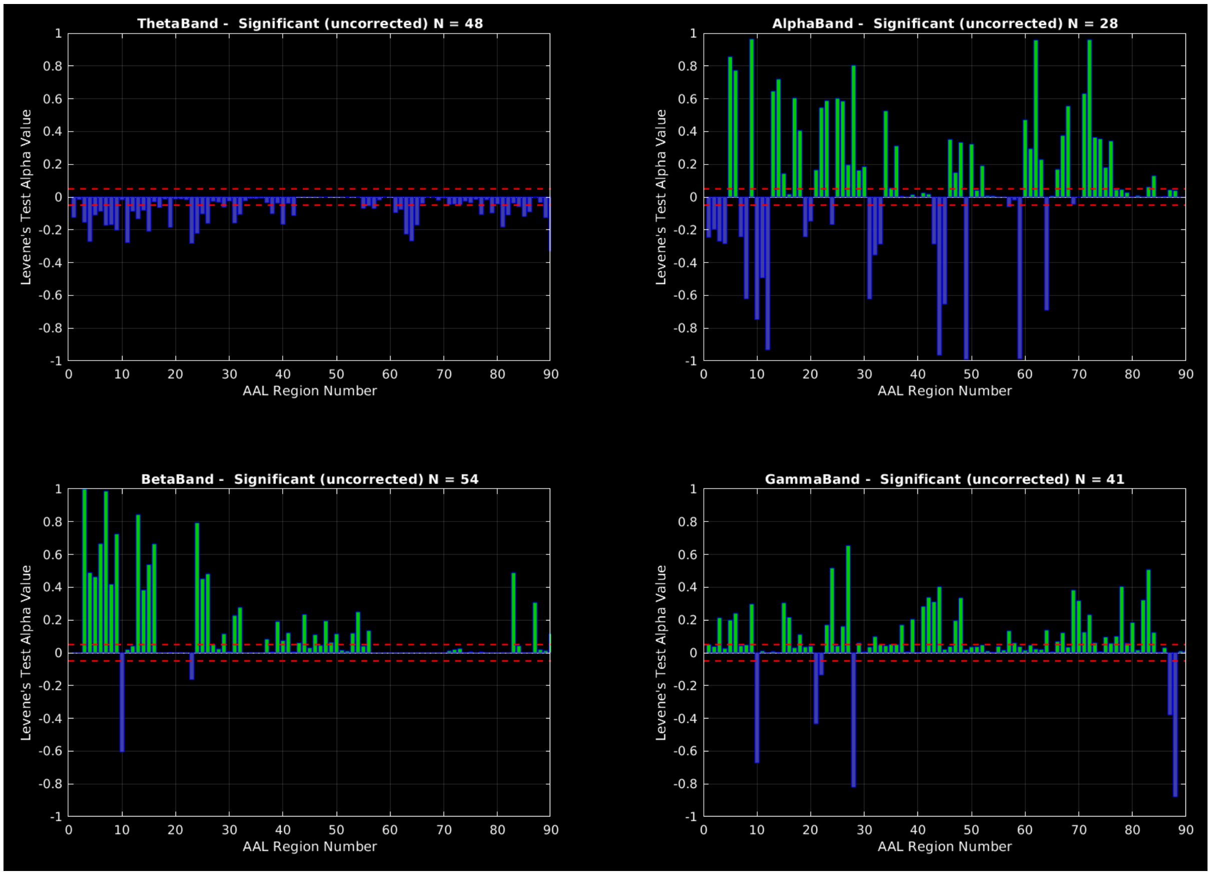

Figure S2. ***Heteroskedasticity of Power with Age.*** *Above, we plot the results of an analysis investigating whether variance of power in older subjects increases relative to younger subjects. The plots above present the alpha value of a series of Levene's tests assessing for inequality of variance between the younger (<18 years) and older (>18 years) subjects within our cohort. The individual bars represent AAL regions, any point falling <0.05 but above 0 indicates that the younger group had significantly (uncorrected) less variance than the older group. Similarly, any bar falling >-0.05 but less than 0 indicates the younger group had significantly (uncorrected) more variance than the older. Some bars were highly significant and so do not appear greatly different from 0.*

Each bar plot describes the results of a single frequency band, each bar representing a different brain region. To ascertain the direction of any effects, the standard deviation of each group was measured and if the variance within the younger group was greater than the older, the alpha value outputted by the Levene's test was multiplied by -1. Any bars in green (above 0) indicate a region with greater variance in the older group and any bars in blue (below 0) indicate greater variance in the younger group. Bars falling inside the dashed red lines indicate this to be a statistically significant (uncorrected) inequality of variance between the two groups.

We found that approximately one half of regions exhibited significant (uncorrected) variance differences between the age groups in the theta, beta, and gamma bands. This proportion fell to approximately one quarter in the alpha band. While the results for the alpha, beta, and gamma

bands suggest there to be both types of outcome (both groups have greater and lesser variance), in the theta band any significant differences in variance arose due to the younger group having more variance than the older.

1.3 Controlling for Signal-to-Noise Ratio when investigating Age-Power Spectral Density Relations

It is possible that the age-related effects seen in our estimation of power spectral density are influenced by noise, arising either from head motion or from signal to noise differences across the age range. As a result, Figure S3 presents two analyses assessing the extent to which age-related changes in SNR or head motion influence our PSD results. Columns A-D present partial-correlations between age and PSD, controlling for SNR measurements derived for each region (based upon the lead fields). The brain plots were generated using these partial correlation values ($r_{partial}$). The graphs in the lower half of these columns present the relation between the original correlation strengths between age and PSD ($r_{original}$) and $r_{partial}$. The lower half of the figure presents our analysis of head motion. Here we plot the relationship between age and maximum head motion (S3-E), average head motion (S3-F), and average head velocity (S3-G). These measurements were derived for each subject and averaged over trials. To investigate the spatial effect of these measures, we again ran partial correlations between PSD and age, controlling for the motion parameter of interest. The results of this analysis are plotted on lower panels of Figure S3. Here, the colour axis shows change in Pearson's r between $r_{partial}$ and $r_{original}$.

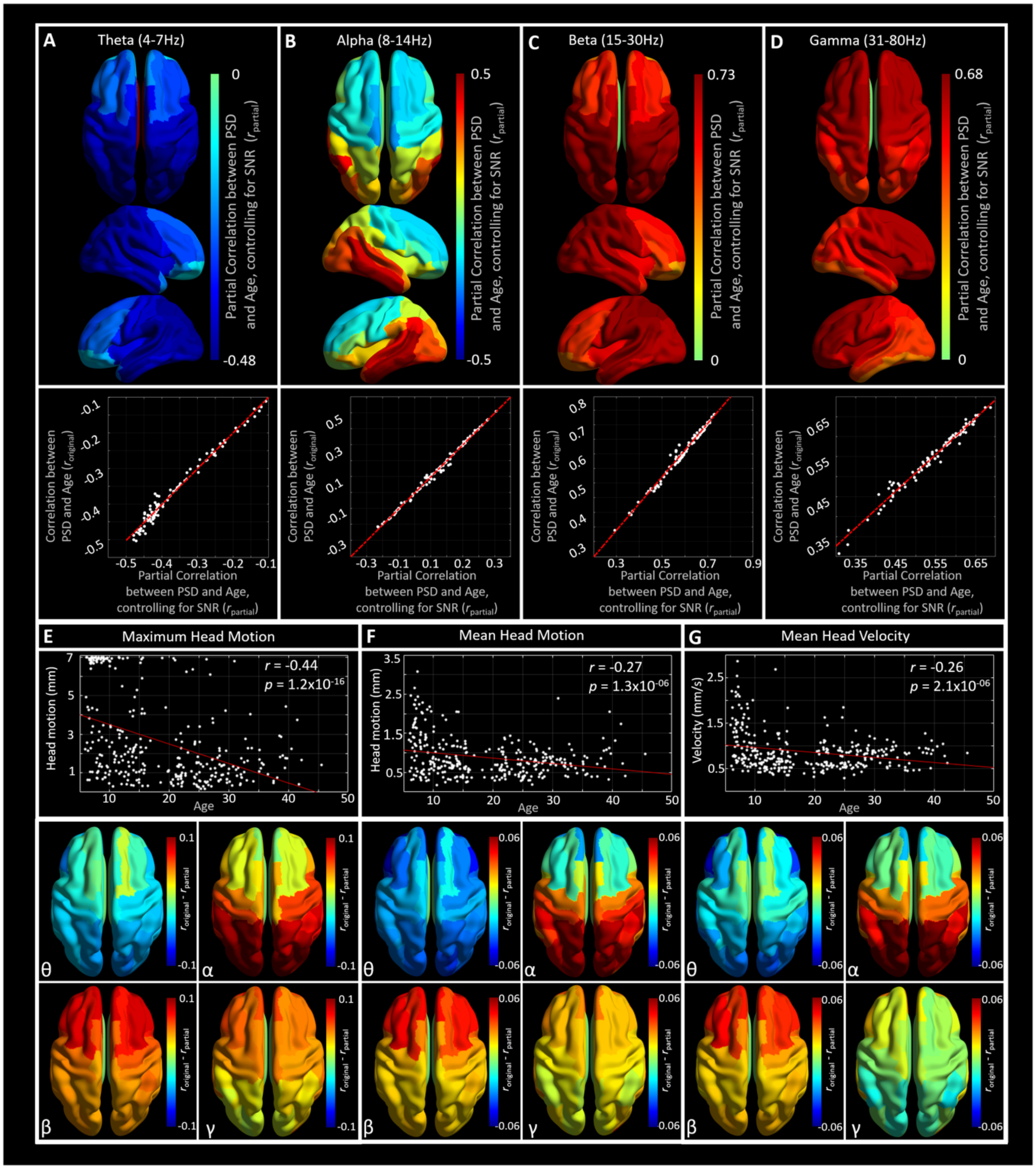

Figure S3 – ***Quantifying the influence of age-related SNR differences on PSD results****. The upper panels A-D present the results of our partial correlations between PSD and Age, controlling for SNR (as derived for Figure 5). Note that the topology and strength of the correlations remains remarkably consistent. The lower panels of A-D present the correlation between the original r values (between age and PSD; $r_{original}$) and the partial correlation results presented above ($r_{partial}$). The lower half of this figure presents results from our analysis assessing the influence of different head motion parameters on PSD results. First, the relation between each parameter and age is plotted. In the lower panels, brain plots show the difference $r_{original} > r_{partial}$ when controlling for the parameter of interest (maximum or average head motion or head velocity). Note that although interesting topological differences occur, the change in r is minimal.*

DOI: 10.1162/netn_a_00077

1.4 Assessing for Sex Bias in wPLI-S curve fitting

While the age distributions of male and female participants are similar, our sample is approximately two thirds male. It is therefore conceivable that our wPLI-S results are driven by male subjects and are not generalisable to females. To investigate this further, we performed an analysis to assess the stability of our fits when the number of males and females were matched. We took a randomly selected subgroup of 116 males and combined with the female participants to form a sex-balanced sample. Using coefficients derived from the full sample we fitted curves and derived a 'real F-statistic'. To assess the statistical significance of the sex-balanced fits, we performed 15,000 permutations where, on each iteration, we randomly shuffled ages, fitted a curve to the shuffled data, and derived a 'null F-statistic'. After the full set of permutations, a null distribution was formed and our significance value calculated as the proportion of nulls falling below our 'real F-statistic'. We repeated this procedure with 100 different sub-samples of males, for each region and frequency band. This entire procedure included 540,000,000 different iterations.

After completion, the iterations produced 100 significance (p) values for each region, for each band. As before, multiple comparisons were controlled using a false discovery rate correction and fits were deemed to be significant if $q<0.05$. To assess the stability of the fits in a sex-balanced sample, the proportion of the 100 iterations that passed this threshold was calculated. If, for example, a fit for a particular region and frequency was found to be significantly characterised by a curve for every iteration of the sex-balanced randomisation, it would have a stability value of 100%. Table S4 displays the results of this procedure, averaged across regions deemed significant in the main analysis (i.e. those plotted on cortical surfaces in Figure 2).

Band	*Mean stability across regions*	*Standard Deviation*
Theta	45.75%	36.8%
Alpha	100%	0%
Beta	98.91%	7.91%
Gamma	57.41%	43%

*Table S4. **Results of our sex-bias analysis.***

Our analysis revealed a lack of bias in alpha and beta band with near perfect stability across iterations. Gamma band performed with near 60% accuracy, meaning that on nearly two thirds of iterations a significant fit was found using the sex balanced data. Theta band performance was

suboptimal in this analysis, and was found to be relatively unstable across runs. This lack of stability suggests that sexual dimorphisms more strongly map onto connectivity within the theta frequency. Furthermore, this may explain the limited number of significantly fitted regions in the primary analysis.

2. Supplementary Visualisation

2.1 Further exploration of the Signal-to-Noise results

Figure S5 presents an extended exploration of our signal to noise ratio results. Here we plot the relation between SNR and Age for two exemplar regions (S5-A) and show the effect of our trial balancing procedure (S5-B).

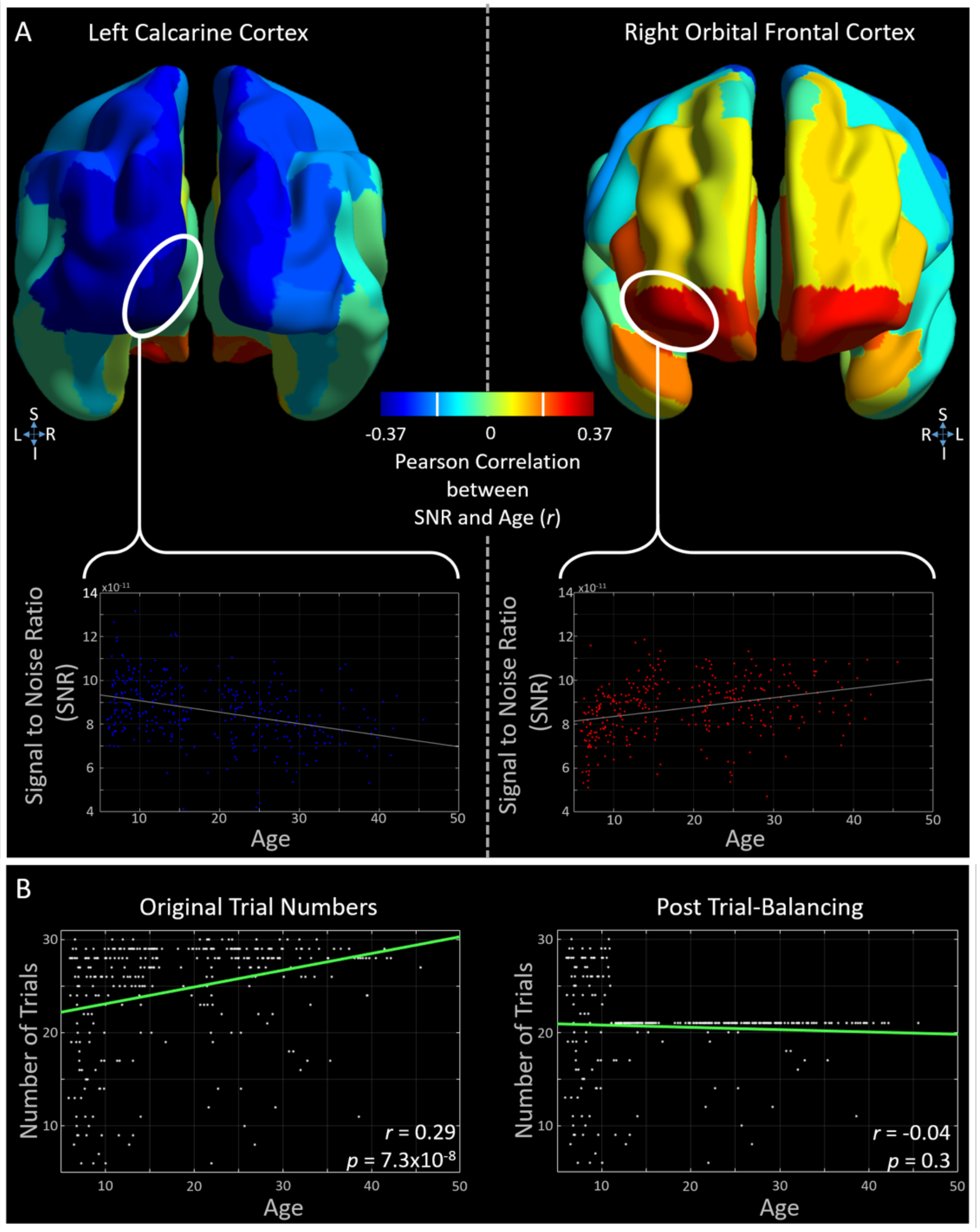

*Figure S5. **Further exploration of age-related confounds.** Panel A plots data for a region of maximal negative correlation (left calcarine cortex) and maximal positive correlation (right orbital frontal cortex). Panel B presents the effect of our trial-balancing procedure by plotting the correlation between number of trials and age first for the original data (left) and second for the same participants following trial balancing (right). Note the absence of significant correlation in the post-trial-balancing figure.*

DOI: 10.1162/netn_a_00077

2.2 Grand-average connectivity at a higher threshold

The circle plots below again present the grand-average wPLI data in Figure 1, but include the strongest 10% of connections (rather than the strongest 5% in Figure 1).

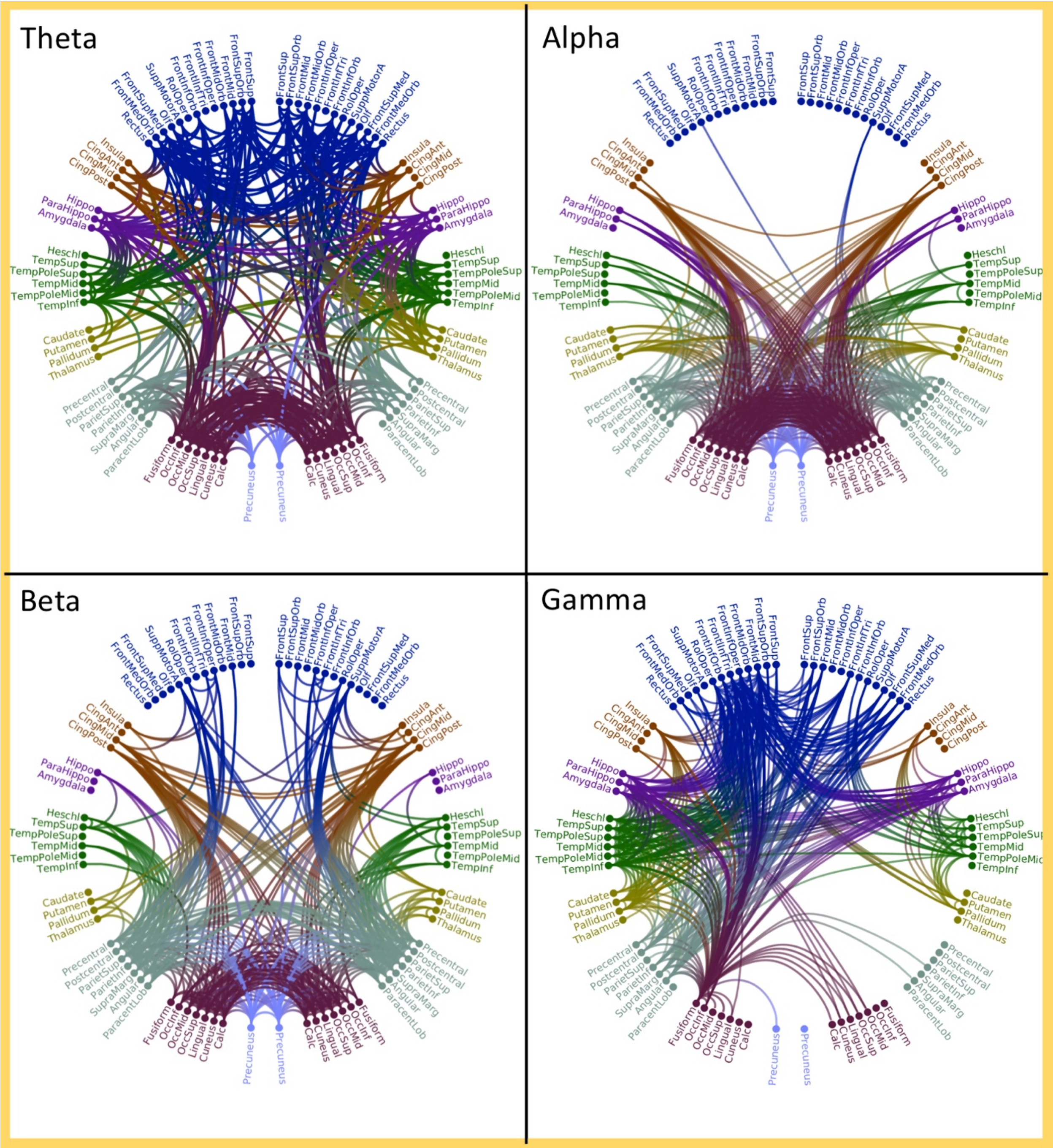

Figure S6. ***Grand-average circle plots at a higher threshold.*** *The circle plots above link to Figure 1, and plot the connections falling within the 90th percentile (rather than the 95th in Figure 1).*

2.3 Videos showing the changing gradient of wPLI-S from childhood to adulthood

To provide an alternative means of interpretation, we present videos showing the gradients of significant model fits for the 45 years our cohort covers.

Video S7
Legend/Caption:
"Video S7 - **Visualising maturational trajectories of connectivity strength: significant fits**. The video presents the curve gradient for each year of age between 6 and 45 years old for the four frequency bands of interest for significant curve fits only. Note that regions with linear trajectories will not change over the course of the video, whereas those with logarithmic or quadratic non-linear trajectories will continue to change."

Video S8
Legend/Caption:
"Video S8 - **Visualising maturational trajectories of connectivity strength – all fits**. The video presents the curve gradient for each year of age between 6 and 45 years old for the four frequency bands of interest for all curves (not only the significantly changing curves). Note that regions with linear trajectories will not change over the course of the video, whereas those with logarithmic or quadratic non-linear trajectories will continue to change."

2.4 Sex and Age Distribution

Below we present a histogram plotting, separately for each sex, the distribution of age in five-year bins.

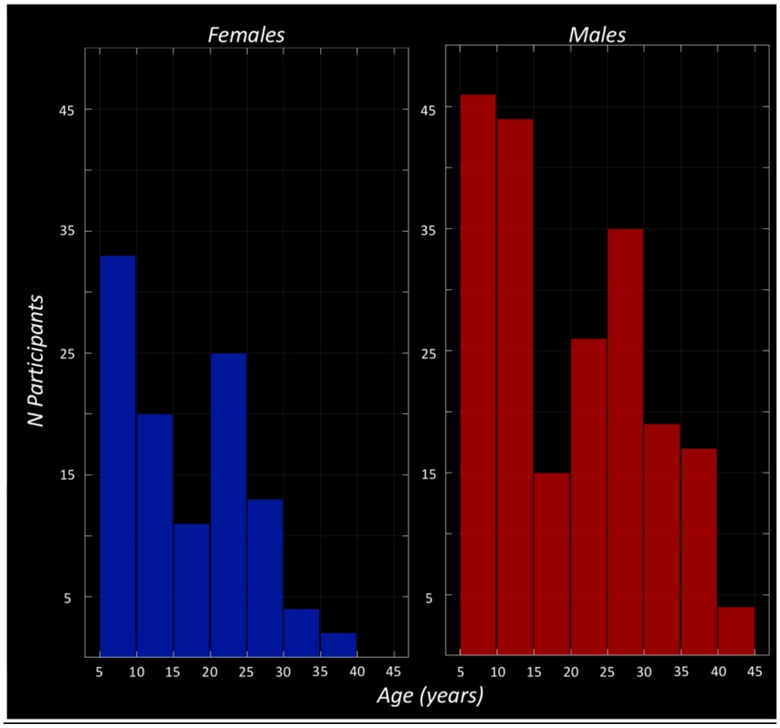

Figure S9. ***Age by Sex Histogram.*** *The histograms plot the age distribution for females (left) and males (right) in 5-year increments. Note the similar distribution across age for both sexes.*

3. Coefficient Table (S10)

We hope that our results may be of use in studies focused on developmental pathology and also hope that other groups, potentially using other modalities, may validate our phase-synchronisation strength results. As such, we present the model coefficients, output by the cross-validation regime, that best characterise the maturational changes in strength of phase synchrony across regions and frequency bands. Note that model type refers to which curve was fit to that region, in that band, where 1 = Linear, 2 = Quadratic and 3 = Logarithmic.

	Theta						Alpha					
Region	Model Type	Coef 1	Coef 2	Coef 3	Coef 4	P-Val	Model Type	Coef 1	Coef 2	Coef 3	Coef 4	P-Val
Precentral_L	3	0.84655	3.05040	0.36788	19.46000	0.00200	3	2.01100	62.50600	2.34160	10.71900	0.00000
Precentral_R	1	0.01413	20.70100	0.00000	0.00000	0.02473	2	-0.00407	0.21454	14.53700	0.00000	0.00000
Frontal_Sup_L	1	0.02000	20.69100	0.00000	0.00000	0.00187	3	2.11720	104.37000	5.69560	9.19480	0.00000
Frontal_Sup_R	1	0.01652	20.63400	0.00000	0.00000	0.01060	2	-0.00311	0.17321	14.28400	0.00000	0.00000
Frontal_Sup_Orb_L	1	-0.00351	21.15900	0.00000	0.00000	0.57760	3	2.55670	14.53400	0.73198	9.84610	0.00000
Frontal_Sup_Orb_R	1	0.00345	21.01100	0.00000	0.00000	0.63940	3	2.27350	155.20000	8.70710	8.08940	0.00000
Frontal_Mid_L	1	0.01502	20.71700	0.00000	0.00000	0.02700	3	2.15340	5.32130	3.86180	11.70900	0.00000
Frontal_Mid_R	1	0.02972	20.25900	0.00000	0.00000	0.00000	2	-0.00300	0.17155	14.07500	0.00000	0.00000
Frontal_Mid_Orb_L	1	0.00308	20.93600	0.00000	0.00000	0.61987	3	2.42220	5.94400	4.31380	10.99600	0.00000
Frontal_Mid_Orb_R	1	0.00721	20.93800	0.00000	0.00000	0.31080	3	1.97930	8.97230	0.80359	11.30300	0.00000
Frontal_Inf_Oper_L	1	0.01503	20.65500	0.00000	0.00000	0.03113	3	2.29940	5.37750	0.47618	11.83800	0.00000
Frontal_Inf_Oper_R	2	0.00050	-0.00844	20.88500	0.00000	0.10347	2	-0.00318	0.19103	14.20100	0.00000	0.00000
Frontal_Inf_Tri_L	1	0.01208	20.67500	0.00000	0.00000	0.06433	3	2.52550	7.29580	16.16600	10.69700	0.00000
Frontal_Inf_Tri_R	1	0.01780	20.58500	0.00000	0.00000	0.00673	3	2.11890	9.52870	18.24400	11.25200	0.00000
Frontal_Inf_Orb_L	1	0.00013	21.02600	0.00000	0.00000	0.98293	2	-0.00320	0.19912	13.82500	0.00000	0.00000
Frontal_Inf_Orb_R	1	0.01818	20.69000	0.00000	0.00000	0.00567	3	2.42460	2.60500	0.19942	12.09600	0.00000
Rolandic_Oper_L	1	0.01285	20.69900	0.00000	0.00000	0.04113	3	2.78970	5.38760	0.54160	11.25900	0.00000
Rolandic_Oper_R	1	0.01752	20.60600	0.00000	0.00000	0.01620	3	2.73790	160.56000	7.86230	7.29940	0.00000
Supp_Motor_Area_L	1	0.01560	20.59200	0.00000	0.00000	0.01600	3	1.70890	13.84100	0.74566	12.53100	0.00013
Supp_Motor_Area_R	1	0.01742	20.57500	0.00000	0.00000	0.00753	3	1.79090	5.19010	0.04537	13.21300	0.00007
Olfactory_L	1	0.00771	20.85200	0.00000	0.00000	0.23293	2	-0.00366	0.21592	14.17700	0.00000	0.00000
Olfactory_R	1	0.01405	20.82500	0.00000	0.00000	0.03907	3	2.88730	486.94000	10.15800	5.44870	0.00000
Frontal_Sup_Medial_L	1	0.01488	20.81200	0.00000	0.00000	0.02267	3	1.81220	7.52930	0.38297	12.11800	0.00000
Frontal_Sup_Medial_R	1	0.00991	20.82000	0.00000	0.00000	0.13753	3	1.57010	10.43800	0.63908	12.36600	0.00000
Frontal_Med_Orb_L	1	0.00315	21.02400	0.00000	0.00000	0.63307	3	2.47470	14.32700	0.02318	10.03500	0.00000
Frontal_Med_Orb_R	1	0.00899	20.90600	0.00000	0.00000	0.19333	2	-0.00252	0.16483	13.99400	0.00000	0.00000
Rectus_L	1	0.00092	21.07800	0.00000	0.00000	0.89060	3	2.71360	9.49580	0.43631	10.22400	0.00000
Rectus_R	1	0.00746	20.94400	0.00000	0.00000	0.27460	2	-0.00318	0.19457	13.93500	0.00000	0.00000
Insula_L	1	0.00650	20.83800	0.00000	0.00000	0.28667	3	2.61960	10.76600	0.26558	10.68100	0.00000

Insula_R	1	0.01626	20.67600	0.00000	0.00000	0.01520	3	2.40590	5.36510	0.35347	11.81700	0.00000
Cingulum_Ant_L	1	0.01850	20.71900	0.00000	0.00000	0.00347	3	2.53820	20.43000	0.81250	10.07700	0.00000
Cingulum_Ant_R	1	0.01495	20.72000	0.00000	0.00000	0.01593	3	2.23080	55.89100	1.08150	9.82540	0.00000
Cingulum_Mid_L	1	0.02009	20.52900	0.00000	0.00000	0.00147	3	2.58530	41.35100	1.29220	9.75890	0.00000
Cingulum_Mid_R	1	0.01764	20.56800	0.00000	0.00000	0.00453	2	-0.00416	0.22664	14.55100	0.00000	0.00000
Cingulum_Post_L	1	0.01887	20.66700	0.00000	0.00000	0.00420	2	-0.00487	0.27677	14.67900	0.00000	0.00000
Cingulum_Post_R	1	0.00963	20.88100	0.00000	0.00000	0.12200	3	3.51240	52.03100	0.66636	7.47130	0.00000
Hippocampus_L	2	-0.00094	0.05441	20.40400	0.00000	0.02887	3	3.34250	3.06260	0.00447	11.50200	0.00000
Hippocampus_R	1	0.00499	20.98100	0.00000	0.00000	0.43027	2	-0.00318	0.21278	14.60600	0.00000	0.00000
ParaHippocampal_L	1	0.00894	21.00700	0.00000	0.00000	0.14860	3	3.51620	20.59300	0.82026	8.26460	0.00000
ParaHippocampal_R	1	0.00896	20.86600	0.00000	0.00000	0.14160	2	-0.00288	0.19733	14.66500	0.00000	0.00000
Amygdala_L	1	0.00687	20.80000	0.00000	0.00000	0.29953	2	-0.00372	0.22498	14.29500	0.00000	0.00000
Amygdala_R	1	0.00845	20.91800	0.00000	0.00000	0.19773	2	-0.00322	0.20274	14.36800	0.00000	0.00000
Calcarine_L	1	0.00924	20.81200	0.00000	0.00000	0.10953	3	3.56100	3.57470	0.08338	12.17300	0.00000
Calcarine_R	1	0.01253	20.74400	0.00000	0.00000	0.04387	2	-0.00457	0.27679	15.08900	0.00000	0.00000
Cuneus_L	1	0.01063	20.78700	0.00000	0.00000	0.07367	3	3.97500	2.31110	0.00748	12.20900	0.00000
Cuneus_R	1	0.00933	20.88900	0.00000	0.00000	0.15307	2	-0.00550	0.31299	14.88900	0.00000	0.00000
Lingual_L	1	0.01569	20.64300	0.00000	0.00000	0.00987	2	-0.00571	0.31892	14.57200	0.00000	0.00000
Lingual_R	1	0.01515	20.74500	0.00000	0.00000	0.01327	3	3.73810	64.43200	7.02000	6.66960	0.00000
Occipital_Sup_L	1	0.00581	20.98100	0.00000	0.00000	0.35520	3	3.98140	21.65500	0.63723	8.15490	0.00000
Occipital_Sup_R	1	0.01622	20.74100	0.00000	0.00000	0.01353	3	3.92920	6.51580	0.15913	10.30700	0.00000
Occipital_Mid_L	3	-0.32908	138.90000	23.54600	22.21500	0.22293	2	-0.00572	0.32915	14.20000	0.00000	0.00000
Occipital_Mid_R	1	0.00388	20.99200	0.00000	0.00000	0.55800	2	-0.00583	0.33033	14.36000	0.00000	0.00000
Occipital_Inf_L	1	0.01553	20.78900	0.00000	0.00000	0.01960	2	-0.00551	0.31985	14.34900	0.00000	0.00000
Occipital_Inf_R	1	0.00461	20.99100	0.00000	0.00000	0.42053	2	-0.00540	0.31225	14.46400	0.00000	0.00000
Fusiform_L	1	0.01926	20.75400	0.00000	0.00000	0.00340	2	-0.00543	0.31622	13.77600	0.00000	0.00000
Fusiform_R	1	0.00761	20.92400	0.00000	0.00000	0.23740	3	3.35890	161.61000	4.32350	5.83360	0.00000
Postcentral_L	1	0.01453	20.57900	0.00000	0.00000	0.02693	2	-0.00432	0.22654	14.74000	0.00000	0.00000
Postcentral_R	1	0.01588	20.59800	0.00000	0.00000	0.01293	3	1.90720	15.95400	0.43669	12.40500	0.00000
Parietal_Sup_L	3	1.00700	2.99690	1.11510	19.28300	0.00053	2	-0.00602	0.32223	14.36000	0.00000	0.00000
Parietal_Sup_R	1	0.00911	20.85200	0.00000	0.00000	0.16767	3	3.04120	48.72600	0.60868	8.98300	0.00000
Parietal_Inf_L	3	0.80222	5.57880	0.12513	19.28900	0.00140	2	-0.00510	0.28780	14.31100	0.00000	0.00000
Parietal_Inf_R	1	0.01364	20.71300	0.00000	0.00000	0.04827	2	-0.00452	0.25695	14.73400	0.00000	0.00000
SupraMarginal_L	1	0.00856	20.82400	0.00000	0.00000	0.18447	2	-0.00462	0.27430	14.17100	0.00000	0.00000
SupraMarginal_R	1	0.01601	20.56500	0.00000	0.00000	0.01527	2	-0.00407	0.22916	14.82400	0.00000	0.00000
Angular_L	3	2.81490	8.16280	287.37000	13.66900	0.00053	2	-0.00568	0.33297	13.85800	0.00000	0.00000

Angular_R	1	0.01173	20.78400	0.00000	0.00000	0.09627	3	3.71450	10.87600	0.20890	9.28490	0.00000
Precuneus_L	1	0.01447	20.75100	0.00000	0.00000	0.01807	2	-0.00529	0.29207	14.72400	0.00000	0.00000
Precuneus_R	1	0.01002	20.86900	0.00000	0.00000	0.11580	3	3.20690	10.95400	0.15092	10.70900	0.00000
Paracentral_Lobule_L	1	0.02080	20.41900	0.00000	0.00000	0.00100	2	-0.00454	0.24006	14.53400	0.00000	0.00000
Paracentral_Lobule_R	1	0.02306	20.42800	0.00000	0.00000	0.00013	2	-0.00499	0.25762	14.50200	0.00000	0.00000
Caudate_L	1	0.02146	20.60300	0.00000	0.00000	0.00040	2	-0.00357	0.21240	14.43400	0.00000	0.00000
Caudate_R	1	0.01774	20.68600	0.00000	0.00000	0.00753	2	-0.00426	0.24156	14.24900	0.00000	0.00000
Putamen_L	1	0.00784	20.77900	0.00000	0.00000	0.21013	3	2.64930	113.36000	2.54620	8.06610	0.00000
Putamen_R	1	0.01369	20.78000	0.00000	0.00000	0.03927	2	-0.00313	0.18960	14.48200	0.00000	0.00000
Pallidum_L	1	0.00930	20.73600	0.00000	0.00000	0.13500	3	2.69280	17.91900	0.56368	10.19300	0.00000
Pallidum_R	1	0.01209	20.81400	0.00000	0.00000	0.07520	2	-0.00351	0.21120	14.45100	0.00000	0.00000
Thalamus_L	1	0.01540	20.65700	0.00000	0.00000	0.01553	2	-0.00460	0.27579	14.12000	0.00000	0.00000
Thalamus_R	1	0.00609	20.93500	0.00000	0.00000	0.33673	3	3.50060	54.19700	0.99934	7.04410	0.00000
Heschl_L	1	0.01657	20.60500	0.00000	0.00000	0.00693	3	3.37080	5.07460	0.24772	10.47700	0.00000
Heschl_R	1	0.01293	20.70000	0.00000	0.00000	0.05587	2	-0.00393	0.24811	14.08700	0.00000	0.00000
Temporal_Sup_L	1	0.01590	20.61400	0.00000	0.00000	0.01453	2	-0.00352	0.22757	14.27200	0.00000	0.00000
Temporal_Sup_R	1	0.01318	20.77700	0.00000	0.00000	0.04887	2	-0.00458	0.27490	13.94600	0.00000	0.00000
Temporal_Pole_Sup_L	1	0.00887	20.76400	0.00000	0.00000	0.16767	3	2.80330	9.41160	0.53297	10.33400	0.00000
Temporal_Pole_Sup_R	1	0.01297	20.78500	0.00000	0.00000	0.05487	3	2.61350	19.28200	1.86240	9.76000	0.00000
Temporal_Mid_L	1	0.01891	20.70100	0.00000	0.00000	0.00553	3	4.00900	15.16000	0.04670	7.61840	0.00000
Temporal_Mid_R	1	0.01524	20.71300	0.00000	0.00000	0.02153	2	-0.00438	0.26974	14.22500	0.00000	0.00000
Temporal_Pole_Mid_L	1	0.01223	20.78700	0.00000	0.00000	0.06320	2	-0.00309	0.19672	14.15600	0.00000	0.00000
Temporal_Pole_Mid_R	1	0.01185	20.87500	0.00000	0.00000	0.06993	3	2.74080	28.10700	0.79073	9.12840	0.00000
Temporal_Inf_L	1	0.02496	20.64300	0.00000	0.00000	0.00007	2	-0.00409	0.26760	13.80700	0.00000	0.00000
Temporal_Inf_R	1	0.01109	20.84500	0.00000	0.00000	0.07973	3	3.39030	0.67432	0.00044	13.54700	0.00000

	Beta						Gamma					
Region	Model Type	Coef 1	Coef 2	Coef 3	Coef 4	P-Val	Model Type	Coef 1	Coef 2	Coef 3	Coef 4	P-Val
Precentral_L	3	0.97485	359.33000	7.10250	6.34090	0.00000	1	-0.01367	5.91360	0.00000	0.00000	0.00020
Precentral_R	3	1.24940	17.72000	2.29910	6.90940	0.00000	1	-0.00137	5.41630	0.00000	0.00000	0.64447
Frontal_Sup_L	3	0.67557	118.46000	7.29260	7.59910	0.00053	3	0.88658	13.41200	14992.00000	1.93450	0.00000
Frontal_Sup_R	3	0.75045	26.35400	4.04160	7.86950	0.00000	3	-0.58761	3409.60000	75.09700	8.28070	0.00000
Frontal_Sup_Orb_L	3	1.72070	2.04260	39.87900	6.41560	0.00013	3	-0.36612	7.51640	7.57600	6.31780	0.01587
Frontal_Sup_Orb_R	1	0.01762	9.30650	0.00000	0.00000	0.00020	1	-0.00141	5.75940	0.00000	0.00000	0.70093
Frontal_Mid_L	3	0.94999	7.56510	0.66388	7.82540	0.00000	2	0.00099	-0.05889	6.61750	0.00000	0.00020
Frontal_Mid_R	1	0.01870	9.47780	0.00000	0.00000	0.00000	2	0.00125	-0.06220	6.22490	0.00000	0.00087
Frontal_Mid_Orb_L	1	0.01732	9.30430	0.00000	0.00000	0.00013	1	-0.00210	5.65570	0.00000	0.00000	0.56393
Frontal_Mid_Orb_R	1	0.01616	9.35550	0.00000	0.00000	0.00000	2	0.00056	-0.03025	5.84960	0.00000	0.09373
Frontal_Inf_Oper_L	1	0.02815	9.35750	0.00000	0.00000	0.00000	1	-0.00813	6.12160	0.00000	0.00000	0.08660
Frontal_Inf_Oper_R	3	1.17730	10.71800	7.59500	7.20730	0.00000	3	-0.37966	0.83180	0.10975	5.98120	0.00747
Frontal_Inf_Tri_L	3	0.95209	3.32490	0.06207	8.17750	0.00000	1	-0.00327	5.95740	0.00000	0.00000	0.46580
Frontal_Inf_Tri_R	3	1.10860	6.59050	16.30800	7.49670	0.00000	2	0.00107	-0.05465	6.29560	0.00000	0.00207
Frontal_Inf_Orb_L	1	0.01768	9.36610	0.00000	0.00000	0.00000	3	-0.53039	2365.60000	22250.00000	8.28840	0.02413
Frontal_Inf_Orb_R	3	0.87199	20.32700	0.87985	7.53380	0.00000	3	-0.27031	0.83950	4.58270	5.90870	0.14580
Rolandic_Oper_L	2	-0.00108	0.06983	9.08150	0.00000	0.00000	1	-0.00960	5.95270	0.00000	0.00000	0.01960
Rolandic_Oper_R	3	1.24630	5.94410	0.62116	7.43760	0.00000	1	-0.00194	5.49920	0.00000	0.00000	0.57847
Supp_Motor_Area_L	3	1.00580	1.44610	0.17759	8.51090	0.00000	3	-0.37037	52.62000	8.42370	6.50250	0.00067
Supp_Motor_Area_R	1	0.02805	9.38570	0.00000	0.00000	0.00000	1	-0.00606	5.42860	0.00000	0.00000	0.01647
Olfactory_L	1	0.02006	9.42980	0.00000	0.00000	0.00000	3	-0.47134	0.26547	2.78150	6.04130	0.04380
Olfactory_R	1	0.02242	9.38830	0.00000	0.00000	0.00000	1	-0.00995	5.88450	0.00000	0.00000	0.01013
Frontal_Sup_Medial_L	3	0.75487	1.48000	0.02029	8.77640	0.00007	2	0.00069	-0.03972	5.96520	0.00000	0.00047
Frontal_Sup_Medial_R	3	0.82236	36.60200	0.34211	7.53420	0.00000	2	0.00111	-0.06166	6.29890	0.00000	0.00000
Frontal_Med_Orb_L	1	0.01633	9.34630	0.00000	0.00000	0.00040	2	0.00057	-0.02836	5.84680	0.00000	0.09007
Frontal_Med_Orb_R	1	0.01575	9.34600	0.00000	0.00000	0.00013	1	-0.00203	5.77520	0.00000	0.00000	0.55160
Rectus_L	1	0.02226	9.25520	0.00000	0.00000	0.00000	2	0.00067	-0.03482	5.88820	0.00000	0.02060
Rectus_R	1	0.02169	9.26680	0.00000	0.00000	0.00000	1	0.00230	5.64630	0.00000	0.00000	0.52220
Insula_L	3	0.90090	8.50890	3.51470	7.94630	0.00000	1	-0.00335	5.79950	0.00000	0.00000	0.37713
Insula_R	3	1.05440	3.93120	0.38027	7.95430	0.00000	2	0.00062	-0.03337	6.04080	0.00000	0.06027
Cingulum_Ant_L	3	0.92638	21.96800	3.15030	7.51440	0.00000	3	-0.29637	2.79080	0.60957	6.00990	0.02153
Cingulum_Ant_R	3	0.84317	9.87590	1.19130	8.03900	0.00000	2	0.00041	-0.02510	5.85980	0.00000	0.02787
Cingulum_Mid_L	3	1.19780	5.09710	0.36624	7.66510	0.00000	3	-0.21665	1.39440	1.01190	5.71240	0.07200
Cingulum_Mid_R	3	1.14950	9.48220	1.77720	7.43290	0.00000	1	-0.00623	5.55700	0.00000	0.00000	0.02960

Cingulum_Post_L	3	1.36010	3.88790	0.17708	7.52080	0.00000	1	-0.00963	5.68100	0.00000	0.00000	0.00100
Cingulum_Post_R	3	1.34980	25.61600	0.81798	6.45540	0.00000	1	-0.00584	5.50950	0.00000	0.00000	0.02920
Hippocampus_L	3	1.50600	3.39030	40.58700	6.88970	0.00000	1	-0.00094	5.78640	0.00000	0.00000	0.82220
Hippocampus_R	3	0.92174	1.46430	0.83691	8.56280	0.00000	3	-1.07320	0.91472	33.47800	7.61180	0.04013
ParaHippocampal_L	1	0.01595	9.48770	0.00000	0.00000	0.00040	1	-0.02037	6.42950	0.00000	0.00000	0.00000
ParaHippocampal_R	1	0.01758	9.48060	0.00000	0.00000	0.00013	3	-0.38680	2.88790	0.12412	6.43670	0.01440
Amygdala_L	3	0.58278	7.40790	7.52120	8.61680	0.00153	1	-0.00361	5.84160	0.00000	0.00000	0.34467
Amygdala_R	3	0.81333	55.75100	5.11860	7.39430	0.00000	2	0.00063	-0.03962	6.34360	0.00000	0.00540
Calcarine_L	3	1.13020	14.72600	0.78539	7.25650	0.00000	1	-0.00769	5.51290	0.00000	0.00000	0.00693
Calcarine_R	3	1.41950	6.89880	7.29000	6.98110	0.00000	1	-0.00663	5.60300	0.00000	0.00000	0.05193
Cuneus_L	3	1.29440	5.33400	0.14455	7.44800	0.00000	1	-0.00324	5.36420	0.00000	0.00000	0.21093
Cuneus_R	2	-0.00131	0.08469	8.94910	0.00000	0.00000	2	0.00052	-0.03764	5.87430	0.00000	0.00000
Lingual_L	3	1.02350	4.97770	0.34625	7.96790	0.00000	1	-0.00408	5.59450	0.00000	0.00000	0.15667
Lingual_R	3	1.23370	2.57280	3.74530	7.89850	0.00000	1	-0.00462	5.58700	0.00000	0.00000	0.13053
Occipital_Sup_L	3	1.99260	68.98900	0.77710	3.85830	0.00000	1	-0.00250	5.47600	0.00000	0.00000	0.43240
Occipital_Sup_R	2	-0.00171	0.10101	8.75140	0.00000	0.00000	3	-0.76502	0.82225	5.15040	6.38450	0.00007
Occipital_Mid_L	2	-0.00211	0.12135	8.46900	0.00000	0.00000	1	-0.00574	5.52250	0.00000	0.00000	0.04053
Occipital_Mid_R	3	1.43640	11.69800	5.56070	6.57600	0.00000	3	-0.57979	7.40930	8.02080	6.68920	0.00007
Occipital_Inf_L	3	1.19810	2.60900	0.95864	7.81330	0.00000	1	-0.00839	5.82240	0.00000	0.00000	0.03087
Occipital_Inf_R	3	1.03110	52.75600	1.81090	6.75580	0.00000	1	-0.00190	5.31120	0.00000	0.00000	0.40733
Fusiform_L	3	1.56540	3.01820	24.18800	6.91960	0.00000	1	-0.00688	6.35310	0.00000	0.00000	0.18787
Fusiform_R	3	0.92173	5.35240	1.42810	8.05840	0.00000	1	-0.00294	5.56760	0.00000	0.00000	0.33533
Postcentral_L	2	-0.00111	0.07240	9.23090	0.00000	0.00000	1	-0.01386	6.03730	0.00000	0.00000	0.00047
Postcentral_R	2	-0.00087	0.05730	9.37350	0.00000	0.00000	1	0.00099	5.31800	0.00000	0.00000	0.71567
Parietal_Sup_L	2	-0.00135	0.09340	8.92710	0.00000	0.00000	1	-0.00212	5.39260	0.00000	0.00000	0.40093
Parietal_Sup_R	2	-0.00187	0.12054	8.64690	0.00000	0.00000	2	0.00046	-0.02358	5.53010	0.00000	0.04827
Parietal_Inf_L	2	-0.00174	0.10297	8.95620	0.00000	0.00000	3	-0.58308	9.23260	5.67580	7.01150	0.00080
Parietal_Inf_R	3	1.62370	1.76230	0.27834	7.77620	0.00000	1	-0.01139	5.65340	0.00000	0.00000	0.00060
SupraMarginal_L	2	-0.00113	0.07825	9.07420	0.00000	0.00000	3	-0.11872	0.63667	0.14334	5.85400	0.45293
SupraMarginal_R	2	-0.00120	0.07998	9.08250	0.00000	0.00000	1	-0.00014	5.29980	0.00000	0.00000	0.95407
Angular_L	2	-0.00111	0.08267	8.96410	0.00000	0.00000	2	0.00103	-0.05657	6.46150	0.00000	0.00920
Angular_R	2	-0.00205	0.12779	8.57410	0.00000	0.00000	2	0.00040	-0.02252	5.66310	0.00000	0.05193
Precuneus_L	3	1.39220	72.64300	5.40370	5.75290	0.00000	2	0.00041	-0.02617	5.72980	0.00000	0.00453
Precuneus_R	3	2.00890	15.74300	2.65250	5.19890	0.00000	1	-0.00454	5.39820	0.00000	0.00000	0.04520
Paracentral_Lobule_L	2	-0.00155	0.09410	8.89090	0.00000	0.00000	1	-0.00473	5.36650	0.00000	0.00000	0.04967
Paracentral_Lobule_R	2	-0.00133	0.08612	8.99340	0.00000	0.00000	1	-0.00308	5.35590	0.00000	0.00000	0.19033

Caudate_L	3	0.88438	7.94810	0.67550	8.06240	0.00000	1	-0.00641	5.77040	0.00000	0.00000	0.07160
Caudate_R	3	1.01580	10.00300	3.04630	7.70390	0.00000	2	0.00042	-0.01911	5.68850	0.00000	0.39013
Putamen_L	3	0.97618	8.54640	11.83700	7.75530	0.00000	1	-0.00430	5.86320	0.00000	0.00000	0.24347
Putamen_R	3	1.04520	7.43170	0.91338	7.70800	0.00000	2	0.00054	-0.02935	6.00950	0.00000	0.08853
Pallidum_L	3	0.77631	6.95580	4.76070	8.29450	0.00000	1	-0.00082	5.75280	0.00000	0.00000	0.81300
Pallidum_R	3	1.00180	9.19570	1.08100	7.72270	0.00000	2	0.00054	-0.03112	6.06020	0.00000	0.05000
Thalamus_L	3	1.02730	71.95900	6.19880	6.74470	0.00000	1	-0.00013	5.52490	0.00000	0.00000	0.96313
Thalamus_R	3	1.02790	5.15130	0.82537	7.92370	0.00000	1	-0.01053	5.71230	0.00000	0.00000	0.00047
Heschl_L	2	-0.00086	0.05970	9.18210	0.00000	0.00000	1	-0.00897	5.92690	0.00000	0.00000	0.02573
Heschl_R	2	-0.00112	0.07527	9.02260	0.00000	0.00000	3	-2.52360	20.04900	2769.20000	14.30900	0.04140
Temporal_Sup_L	2	-0.00077	0.05499	9.19980	0.00000	0.00000	1	-0.01038	6.03210	0.00000	0.00000	0.02367
Temporal_Sup_R	3	1.59490	2.01350	11.07200	7.25170	0.00000	1	-0.00176	5.45540	0.00000	0.00000	0.61707
Temporal_Pole_Sup_L	1	0.01262	9.49960	0.00000	0.00000	0.00413	1	0.00066	5.67580	0.00000	0.00000	0.85393
Temporal_Pole_Sup_R	3	0.94052	8.98590	0.60362	7.71560	0.00000	3	-0.17430	14.89000	9.82220	6.04540	0.28847
Temporal_Mid_L	1	0.02420	9.36270	0.00000	0.00000	0.00000	1	-0.00474	5.90000	0.00000	0.00000	0.31147
Temporal_Mid_R	3	1.23240	23.21700	1.61460	6.66990	0.00000	1	-0.00297	5.55520	0.00000	0.00000	0.44053
Temporal_Pole_Mid_L	1	0.01406	9.45400	0.00000	0.00000	0.00147	1	-0.00486	5.74950	0.00000	0.00000	0.15787
Temporal_Pole_Mid_R	3	0.80640	9.83140	1.16810	7.92970	0.00000	1	-0.00992	5.92500	0.00000	0.00000	0.01400
Temporal_Inf_L	2	-0.00070	0.05087	9.14740	0.00000	0.00000	2	0.00103	-0.06661	6.73860	0.00000	0.00000
Temporal_Inf_R	3	0.88805	3.75710	0.21203	8.17140	0.00000	1	-0.00823	5.67810	0.00000	0.00000	0.01220
